# Genome-wide identification and expression profiling of *auxin response factor *(*ARF*) gene family in maize

**DOI:** 10.1186/1471-2164-12-178

**Published:** 2011-04-07

**Authors:** Hongyan Xing, Ramesh N Pudake, Ganggang Guo, Guofang Xing, Zhaorong Hu, Yirong Zhang, Qixin Sun, Zhongfu Ni

**Affiliations:** 1 State Key Laboratory for Agrobiotechnology and Key Laboratory of Crop Heterosis and Utilization (MOE), Key Laboratory of Crop Genomics and Genetic Improvement (MOA), Beijing Key Laboratory of Crop Genetic Improvement, China Agricultural University, Beijing, 100193, China; 2 National Plant Gene Research Centre (Beijing), Beijing, 100193, China; 3 National Maize Improvement Center, Beijing, 100193, China

## Abstract

**Background:**

Auxin signaling is vital for plant growth and development, and plays important role in apical dominance, tropic response, lateral root formation, vascular differentiation, embryo patterning and shoot elongation. Auxin Response Factors (ARFs) are the transcription factors that regulate the expression of auxin responsive genes. The *ARF *genes are represented by a large multigene family in plants. The first draft of full maize genome assembly has recently been released, however, to our knowledge, the *ARF *gene family from maize (*ZmARF *genes) has not been characterized in detail.

**Results:**

In this study, 31 maize (*Zea mays *L.) genes that encode ARF proteins were identified in maize genome. It was shown that maize *ARF *genes fall into related sister pairs and chromosomal mapping revealed that duplication of *ZmARFs *was associated with the chromosomal block duplications. As expected, duplication of some *ZmARFs *showed a conserved intron/exon structure, whereas some others were more divergent, suggesting the possibility of functional diversification for these genes. Out of these 31 *ZmARF *genes, 14 possess auxin-responsive element in their promoter region, among which 7 appear to show small or negligible response to exogenous auxin. The 18 *ZmARF *genes were predicted to be the potential targets of small RNAs. Transgenic analysis revealed that increased miR167 level could cause degradation of transcripts of six potential targets (*ZmARF3*, *9*, *16*, *18*, *22 *and *30*). The expressions of maize *ARF *genes are responsive to exogenous auxin treatment. Dynamic expression patterns of *ZmARF *genes were observed in different stages of embryo development.

**Conclusions:**

Maize *ARF *gene family is expanded (31 genes) as compared to *Arabidopsis *(23 genes) and rice (25 genes). The expression of these genes in maize is regulated by auxin and small RNAs. Dynamic expression patterns of *ZmARF *genes in embryo at different stages were detected which suggest that maize *ARF *genes may be involved in seed development and germination.

## Background

Auxin signaling plays a vital role in plant growth and development processes like, in apical dominance, tropic responses, lateral root formation, vascular differentiation, embryo patterning and shoot elongation [1]. At the molecular level, most of these processes are controlled by the auxin-response genes [2,3], and auxin responsiveness is conferred to these genes by conserved promoter elements, including TGA-element (AACGAC), AuxRR-core (core of the auxin response region, GGTCCAT) and AuxRE (auxin response element, TGTCTC). Among these, the AuxRE promoter elements are bound and activated by a plant-specific transcription factors which are called as Auxin Response Factors (ARFs) [4-8]. An ARF protein contains a DNA-binding domain (DBD) in the N-terminal region, a middle region that functions as an activation domain (AD) or repression domain (RD) [9,10], and a carboxyl-terminal dimerization domain (CTD) that are similar to those found in the C terminus of Aux/IAAs, which is a protein-protein interaction domain that mediates the homo- and hetero- dimerization of ARFs and also the hetero-dimerization of ARF and Aux/IAA proteins [9-14].

It has been reported that, the ARF proteins are encoded by a large gene family, with 23 and 25 members in *Arabidopsis *and rice, respectively [15,16]. Expression analysis suggested that these genes are, in general, transcribed in a wide variety of tissues and organs, with an exception of *ARF *gene cluster on *Arabidopsis *chromosome 1, which appears to be restricted to embryo genesis/seed development [15]. Classical genetic approaches have led to the identification of *ARF *gene functions in plant growth and development. For example, *arf *mutations caused the change in gynoecium patterning (*AtARF3*) [17-19], impaired hypocotyls response to blue light, growth and auxin sensitivity (*AtARF7*) [20-24], formation of vascular strands and embryo axis formation (*AtARF5*) [25], suppression of hookless phenotype and hypocotyl bending (*AtARF2*) [26-28], hypocotyl elongation, and auxin homeostasis (*AtARF8*) [29,30]. Moreover, the mutants of *AtARF *sister pairs generally exhibit a much stronger phenotype than that of single mutants, suggesting that closely related *AtARFs *have somewhat redundant roles in *Arabidopsis *[15]. In rice, antisense phenotype of *OsARF1 *gene showed stunted growth, low vigor, curled leaves and sterility, suggesting that the gene is essential for vegetative and reproductive development [31].

Genetic divergence between *Arabidopsis *and rice *ARF *gene family investigated by genome-wide analysis revealed that most of the rice *OsARFs *are related to *Arabidopsis ARFs *and fall into sister pairs as in *Arabidopsis *[16,32]. The first assembly of maize genome sequence has recently been published [33], however, to the best of our knowledge, the maize *ARF *gene family (*ZmARF *genes) has not been characterized in detail. In this article, we provide detailed information on the genomic structures, chromosomal locations, sequence homology and expression patterns of 31 maize *ARF *genes. In addition, the phylogenetic relationship between *ARF *genes in *Arabidopsis*, rice and maize were also compared, which will help future studies for elucidating the precise roles of *ZmARFs *in maize growth and development.

## Results

### **Identification and chromosomal **localization **of maize *ARF *genes**

Extensive searches of public and proprietary transcript and genomic databases, by using all previously reported ARF proteins from other species as BLAST queries, identified a total of 31 maize ARF genes that have complete sequences in respective bacterial artificial chromosome (BAC) clones. Among these full-length coding sequences of 13 *ZmARF *genes (*ZmARF1*, *3*, *9*, *10*, *12*, *16*, *18*, *20*, *22*, *24*, *25*, *27 *and *30*) were further confirmed by RT-PCR amplification, cloning and sequencing (Additional file 1). The full length coding sequences of the *ZmARF *genes ranged from 1389 bp (*ZmARF31*) to 3450 bp (*ZmARF20*) with the deduced proteins of 462 to 1149 amino acids (Table 1).

**Table 1:** *ARF *gene family in Maize

Gene Name^a^	ORF length (bp)^b^	Deduced polypeptide^c^			Chr. Locus^d^	Genomic Locus^e^			EST^g^	Physical Block^h^	Block Pairs^i^
							
		Length (aa)	MW (kDa)	PI		BAC	**GenBank Accessions**, DNA	Nearest Marker^f^			
*ZmARF1*	3261	1086	120.15	6.25	1.06	AC208531	HM004516	bnlg1057	CO533792		
*ZmARF2*	2046	681	74.53	7.93	1.08	AC194848	HM004517	umc1096	BT060467	45-49	10La
*ZmARF3*	2451	816	90.85	6.38	2.01	AC191413	HM004518	umc2046	AY110452	68-82	4L
*ZmARF4*	2808	935	102.78	6.12	2.01	AC204518	HM004519	umc1622	EE039613	68-82	4L
*ZmARF5*	1542	513	55.44	6.40	2.03	AC200303	HM004520	umc1021	BT066632	68-82	4L
*ZmARF6*	1974	657	72.94	6.14	2.04	AC190503	HM004521	umc1089	BT067327		
*ZmARF7*	2061	686	76.70	6.29	3.00	AC190684	HM004522	bnlg108	EC885481		
*ZmARF8*	2124	707	75.35	7.10	3.01	AC184124	HM004523	phi049	EU965402		
*ZmARF9*	2646	881	97.03	6.01	3.04	AC193407	HM004524	AI881370	EE037883		
*ZmARF10*	2400	799	89.22	6.25	3.06	AC193433	HM004525	bnlg1176	BT055015	125-151	1L
*ZmARF11*	2067	688	74.98	7.28	3.07	AC210193	HM004526	pco114882	DR820421	125-151	1L
*ZmARF12*	2127	708	77.95	7.11	3.08	AC202124	HM004527	umc1690	EU947189		
*ZmARF13*	2553	850	93.13	7.01	4.03	AC197426	HM004528	umc2199	BT069005		
*ZmARF14*	2019	672	74.84	6.25	4.05	AC208514	HM004529	umc1103	EE174911		
*ZmARF15*	2136	711	75.93	7.23	4.06	AC205511	HM004530	bnlg1755	BT087991		
*ZmARF16*	2718	905	100.23	6.10	4.09	AC195460	HM004531	umc1143	EU955385		
*ZmARF17*	1935	644	70.95	6.97	5.03	AC184866	HM004532	umc2352	BT066347	212-219	10La
*ZmARF18*	2742	913	100.89	6.54	5.03	AC196992	HM004533	umc1315	EE176799		
*ZmARF19*	2151	716	77.51	7.14	5.03	AC207656	HM004534	umc1610	AY105182		
*ZmARF20*	3450	1149	127.47	6.17	5.03	AC194218	HM004535	umc1154	EE290325		
*ZmARF21*	2097	698	74.99	8.26	6. 02	AC197562	HM004536	umc1656	BT083638		
*ZmARF22*	2778	925	102.23	6.44	6. 02	AC195867	HM004537	bnlg2151	CA272068		
*ZmARF23*	2043	680	73.94	6.86	6. 06	AC187896	HM004538	umc1103	BT067427		
*ZmARF24*	2211	736	80.45	7.84	6. 07	AC204859	HM004539	umc1350	AY109838		
*ZmARF25*	2406	801	89.75	6.47	8.06	AC203318	HM004540	umc1161	BT067605	324-366	1L
*ZmARF26*	2061	686	74.34	7.10	8.09	AC208613	HM004541	umc1638	BT087971	324-366	1L
*ZmARF27*	3162	1053	116.73	6.67	9.01	AC211017	HM004542	umc2335	CD439516		
*ZmARF28*	2442	813	89.69	7.14	10.03	AC190927	HM004543	umc1863	BT066544		
*ZmARF29*	2838	945	103.82	6.31	10.07	AC201888	HM004544	pco137895	CO441386	411-420	4L
*ZmARF30*	2430	809	90.30	6.24	10.07	AC200880	HM004545	pco137999	EC901065	411-420	4L
*ZmARF31*	1389	462	50.55	5.41	10.07	AC190828	HM004546	umc1038	BT067850	411-420	4L

**^a ^**Systematic designation given to maize ARFs in this work.

**^b ^**Length of open reading frame in base pairs.

**^c ^**The number of amino acids, molecular weight (kilodaltons), and isoelectric point (p*I*) of the deduced polypeptides.

**^d ^**Location of the *ZmARF *genes to the Chromosome bins.

**^e ^**BAC name, DNA accession number where the *ZmARF *gene is present.

**^f ^**Nearest marker to the *ZmARF *gene.

**^g ^**Representative EST/cDNA in GenBank corresponding to *ZmARF *gene.

**^h ^**Location of the *ZmARF *genes to the physical blocks [32].

**^I ^**Location of the *ZmARF *genes to the block pairs [32]. Six *ZmARF *sister pairs are mapped on the same duplicated chromosomal blocks: *ZmARF2 *and *17 *in 10La, *ZmARF3 *and *30 *in 4L, *ZmARF4 *and *29 *in 4L, *ZmARF5 *and *31 *in 4L, *ZmARF10 *and *25 *in 1L, *ZmARF11 *and *26 *in 1L.

**Figure 1:**
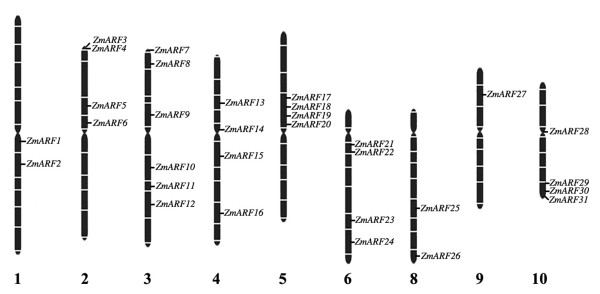
**Chromosomal distribution of *ARF *genes in maize**. Constrictions on the chromosomes (vertical bar) indicate the position of centromeres. The chromosome numbers (except for Chromosome 7) are indicated at the bottom of each chromosome image.

The nearest genetic markers for each *ARF *gene were determined from maize BAC contigs and positioned on maize genetic map. It was found that these 31 *ZmARFs *were mapped on 9 out of 10 maize chromosomes, except for chromosome 7. Six *ZmARFs *were present on chromosome 3; 4 on chromosomes 2, 4, 5, 6 and 10; 2 on chromosomes 1 and 8; only one on chromosome 9 (Table 1, Figure 1). In addition, six *ZmARF *sister pairs were mapped on the same duplicated chromosomal blocks that has been described previously [34] (Table 1).

The predicted molecular weights of the 31 deduced ZmARF proteins ranged from 50.55 kDa (ZmARF31) to 127.47 kDa (ZmARF20) (Table 1). Pair-wise analysis of ZmARF protein sequences indicated that the overall identity fell in a range within 1.9% (between ZmARF28 and ZmARF31) to 54.3% (between ZmARF11 and ZmARF26) (Additional file 2). Moreover, multiple protein sequence alignments were performed using the CLUSTAL_X program to examine structural features of these 31 maize *ARF *genes. The results revealed that all the ZmARF proteins contained a highly conserved region of about 320 amino acid residues in their N-terminal portion corresponding to the DNA-binding domain (DBD) of *Arabidopsis *ARF family. Except ZmARF 5 and 31, the other ZmARF proteins contained a carboxyl-terminal domain (CTD) related to domains III and IV found in Aux/IAA proteins (Additional file 3).

It has been reported that the middle region of ARFs function as activation or repression domains [35]. Transfection assays with plant protoplasts indicated that AtARF1, 2, 3, 4, and 9 are repressors [35,36], among which AtARF1 contain middle region rich in proline (P), serine (S) and threonine (T). AtARF5, 6, 7, 8 and 19, which contain middle region rich in glutamine (Q), are activators [37,38]. The detailed sequence analysis of all 31 deduced ZmARF proteins revealed that PST rich middle regions were found in ZmARF6, 10, 13, 14, 25 and 28, indicating that these genes are more likely to act as repressors. While Q rich regions were found in ZmARF1, 3, 9, 16, 18, 19, 22, 27 and 30, implying that these genes are probable transcriptional activators (Additional file 3).

### Phylogenetic analysis and genomic structure of ZmARFs

**Figure 2:**
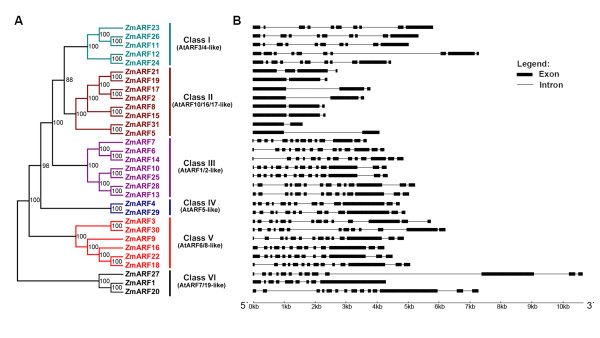
**Phylogenetic relationship among the ZmARF proteins and exon-intron organization of the *ZmARF *genes**. A: Phylogenetic relationship among the maize ARF proteins. The unrooted tree was generated using MRBAYES 3.1.2 program by Bayesian method and the bootstrap test was carried out with 20,000 iterations; Numbers on the nodes indicate clade credibility values; Gene classes were indicated with different colors. B: Exon-intron organization of corresponding *ZmARF *gene. The exons and introns are represented by boxes and lines, respectively.

Bayesian phylogenetic analysis was performed and the 31 ZmARF proteins were classified into six classes: Class I (AtARF3/4-like), II (AtARF10/16/17-like), III (AtARF1/2-like), IV(AtARF5-like), V(AtARF6/8-like) and VI(AtARF7/19-like), with each class containing 5, 8, 7, 2, 6 and 3 members, respectively (Figure 2A). It was worthy to note that in the joint phylogenetic tree most of the ZmARF proteins fell as related sister pairs (Figure 2A), *viz*. ZmARF2 and 17, 3 and 30, 4 and 29, 5 and 31, 6 and 14, 8 and 15, 10 and 25, 12 and 24, 13 and 28, 19 and 21, or triplets (ZmARF11, 23 and 26; 1, 20 and 27) and quadruplets in the case of ZmARF9, 16, 18 and 22.

The intron/exon structures of *ZmARF *genes were determined by alignment of cDNA to genomic sequences. This sequence analysis revealed that introns were found in coding sequences of all the *ARF *genes and the number of exons varied from 2 to 14 (Figure 2B). As expected, most *ZmARF *genes in the same sister pair or triplets showed similar distribution of intron/exon, whereas the others were more divergent in genomic structure, suggesting that these sister pair genes lies in duplicated genomic regions (Figure 2B).

**Figure 3:**
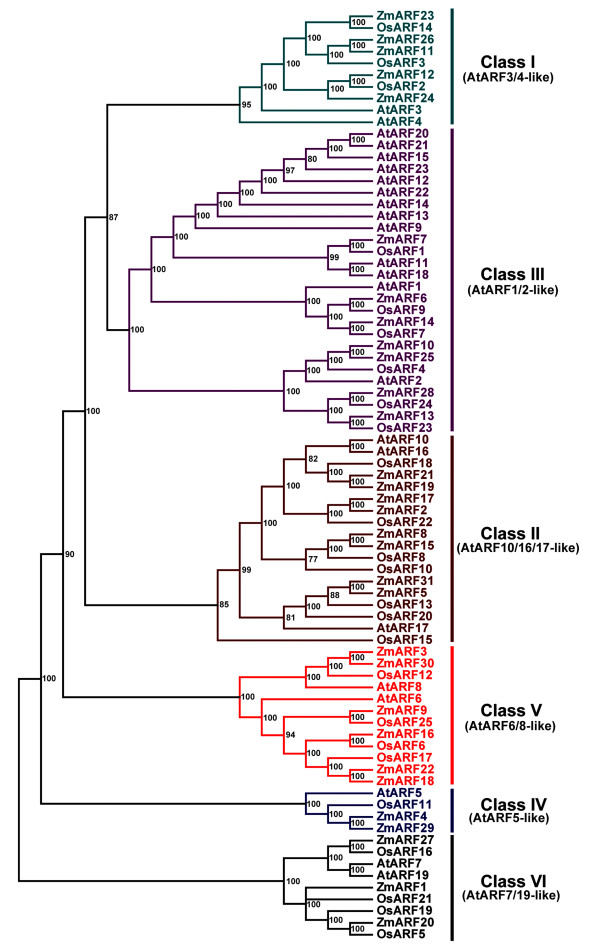
**Phylogenetic analysis of maize, rice and *Arabidopsis *ARF proteins**. The protein sequences of *Arabidopsis *and rice auxin response factors were obtained from the TIGR database, and phylogenetic analysis was performed with MRBAYES 3.1.2 program by Bayesian method and the bootstrap test was carried out with 1,000,000 iterations; Numbers on the nodes indicate clade credibility values; The gene classes were indicated with the same categories and colors as in Figure 2.

The annotated *ARF *gene family in *Arabidopsis *and rice enabled us to determine the phylogenetic relationship between dicot and monocot ARF proteins. A phylogenetic tree constructed using the protein sequences of 31 ZmARFs, 25 OsARFs and 23 AtARFs depicted that all of these 79 ARF proteins were also divided into six classes (Figure 3).

### Prediction of potential targets for small RNA

Previous studies have revealed that several *ARF *genes are targets for small RNAs. For example, *AtARF6 *and *8 *are miR167 targets [39-41] while *At*ARF*10*, *16*, and *17 *are miR160 targets [40,42,43]. *AtARF2*, *3 *and *4 *are targets for *trans*-acting-small interfering RNA 3 (*TAS3 ta-siRNA*) [40,44]. In this study, putative small RNA target sites were searched by using the miRanda software. 18 out of 31 *ZmARF *genes were predicted to be the potential targets of small RNA and the number of target genes for miR160, miR167, *TAS3 ta-siRNA *was 7, 6 and 5, respectively (Additional file 4).

**Figure 4:**
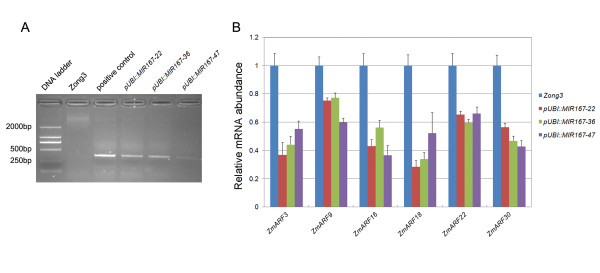
**Relative mRNA abundances of *AtARF6/8*-like *ZmARFs *in wild-type and *pre*-*MIR167b *overexpression lines**. A. Identification of *pre-MIR167b *overexpressing transgenic lines by DNA-based *bar *gene amplification. Amplification products were separated by agarose gel electrophoresis. B. Quantitative real-time RT-PCR analysis was performed by using cDNAs from roots of 8-day-old seedling of *MIR167b *transgenic lines (*pUBI::MIR167b-22*, *-36 *and *-47*) and wild type (Zong3).

To determine whether the increased miR167 level could cause the degradation of transcripts of the six potential targets (*ZmARF3*, *9*, *16*, *18*, *22 *and *30*), we generated miR167 overexpressing Zong3 lines and the presence of transgene was confirmed by PCR amplifications of *bar *gene (Figure 4A). We then analyzed mRNA levels of these genes in roots of 8-day-old seedling in wild type and miR167 overexpressing Zong3 lines (Figure 4B). The results of real-time RT-PCR exhibited that mRNA abundance of *ZmARF3*, *9*, *16*, *18*, *22 *and *30 *in three independent transgenic lines decreased markedly as compared with wild type, indicating that post-transcriptional regulation by small RNAs may play an important role in regulating the expression of *ZmARF *genes (Figure 4B).

### Auxin inducibility and promoter motif prediction of *ZmARF *genes

**Figure 5:**
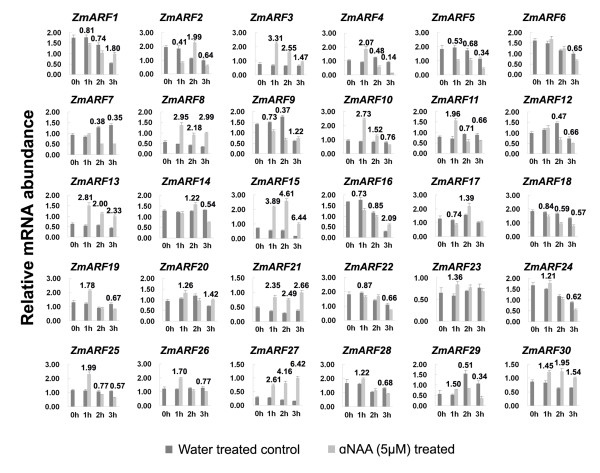
**Fold induction of *ZmARF *genes response to exogenous auxin stimuli**. For maize inbred line B73, 8-day-old primary roots were harvested after0, 1, 2 and 3 hours of incubation in 5 μM αNAA and distilled water. Relative mRNA abundance of each gene was normalized with *β-Actin *gene, with the exception of *ZmARF31 *(No signal detected in this tissue). Paired *t*-test was used to determine the significance of differences of relative mRNA abundance between auxin treatment and water control at each time point, and fold induction with significant difference was listed at the top of column.

To determine the response of *ZmARF *genes to exogenous auxin stimuli, their expression patterns in seedling roots at 1, 2 and 3 hours after 5 μM αNAA treatment were investigated using real-time RT-PCR and fold induction relative to water-treated controls for each time point were calculated. This analysis revealed that, with an exception of *ZmARF31*, all other genes were expressed in seedling roots, among which 7 genes (*ZmARF3*, *8*, *13*, *15*, *21, 27 *and *30*) were up-regulated and 2 genes (*ZmARF5 *and *18*) were down-regulated by exogenous auxin treatment across all time points. In addition 7 genes (*ZmARF4*, *11*, *19*, *24*, *25*, *26 *and *29*) were up-regulated by auxin after 1 h treatment but down-regulated later. In contrast, 3 genes (*ZmARF1*, *9 *and *16*) were down-regulated over the first 2 h of treatment but up-regulated after 3 h of auxin treatment. The other 11 *ZmARF *genes also displayed diverse expression pattern in response to auxin treatment, indicating the complexity of auxin-regulated gene expression of *ZmARF *genes (Figure 5). Interestingly, the relative mRNA abundance of some ARF genes, for example *ZmARF1, 7 and 16*, were also altered dramatically in water treatment, indicating that these genes may be responsive to water logging.

Putative promoter sequences (1500 bp upstream the 5'UTR region) of ZmARFs were obtained from the draft maize genome sequence. Database search of plant promoters (PlantCARE) [45] detected one or more auxin response elements in some of these putative promoters. Notably, three auxin response elements (2 TGA-elements and 1 AuxRE) were detected in the promoter region of *ZmARF15 *gene, consistent with its significantly increased mRNA accumulation following auxin treatment. However, out of 31 *ZmARFs *genes, auxin response elements were detected only in promoter regions of 14 *ZmARFs *genes (Additional file 5). Thus, the relationship between auxin-response elements and auxin inducibility of *ZmARF *genes remains unclear and need to be further investigated.

### Expression profiling of *ZmARF *genes in embryos during seed development and germination

**Figure 6:**
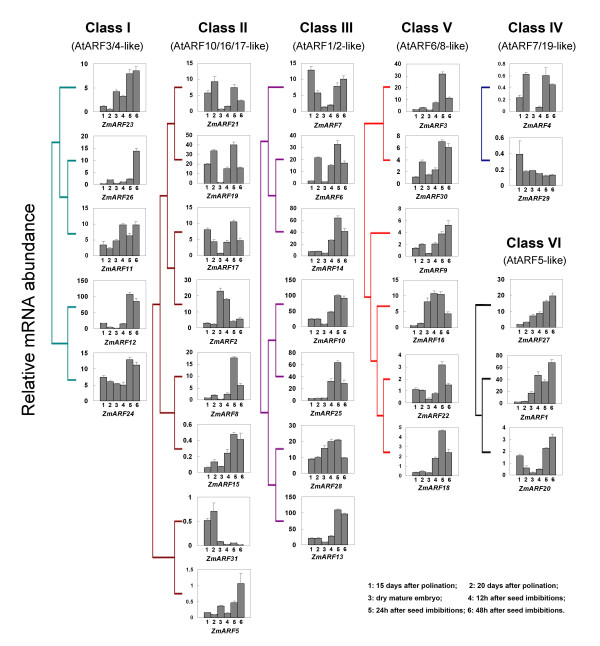
**Expression profiling of *ZmARF *genes in embryos during seed development and germination**. For maize inbred line B73, seed embryos of 15 and 20 days after pollination were harvested from greenhouse-grown plants which were planted in a sand/peat under 15 h of light (25°C) and 9 h of dark (20°C) conditions, and embryos were detached from seeds at 0, 12, 24 and 48 h after imbibition in a chamber at 25°C with 12 h light/dark cycle (12 h day/12 h night).

Recently, functional analysis has revealed that some *ARF *genes in Arabidopsis play important role in seed development and germination [28,46]. As a result, we focused on the study of expression patterns of *ZmARF *genes in embryos during seed development and germination (Figure 6). Expressions of all 31 *ZmARF *genes were detected but they exhibited dynamic expression patterns, of which 16 genes (51.6%) showed peak expression in embryos at 24 h after imbibition. In addition, the expression patterns of duplicated *ZmARF *genes also varied considerably. For example, the expression level of *ZmARF1 *gene was much higher than that of its sister gene *ZmARF20 *and the time point of peak expression was also different for *ZmARF2 and ZmARF17*. It is worthy to note that the relative mRNA abundances of 8 *ZmARF *genes in dry mature embryos were higher than in immature embryos which further increased during seed germination (Figure 6). We observed that the mRNA accumulation of *ZmARF2 *gene peaked in dry mature embryo but declined after seed imbibition (Figure 6). RT-PCR analysis of embryos (15d after pollination) and two vegetative tissues (8-day-old seedling leaves and roots) detected transcripts of *ZmARF *genes with an exception of *ZmARF31 *in 8-day-old seedling leaves and roots (Additional file 6).

## Discussion

### Characterization of an expanded *ARF *gene family in maize

It is believed that the long evolutionary periods experienced by a particular organism is the cause of having multiple members in the specific gene family [16,47]. Several rounds of whole genome duplication have been reported in the maize genome [48]. In this study, we identified and characterized 31 maize *ARF *genes through genome-wide analysis, suggesting that maize *ARF *gene family was expanded compared to *Arabidopsis *(23) and rice (25) [15,16]. Phylogenetic analysis revealed that maize *ARF *gene family contained ten sister pairs, two triplets and one quadruplet. However, none of these pairs were genetically linked to each other on their corresponding chromosomal locations. On the other hand, all closely linked *ZmARF *loci such as *ZmARF3 *and *ZmARF4 *on chromosome 2; *ZmARF17*, *18*, *19 *and *20 *on chromosome 5 and *ZmARF29*, *30 *and *31 *on chromosome 10 were not paired together into the same sister groups. Moreover, at least six *ZmARF *sister pairs mapped on the same duplicated chromosomal blocks (Table 1) [34]. Thus, the expansion of maize *ARF *gene family could be explained by the ancient tetraploid ancestry of maize, in which genome duplication occurred after divergence from the common ancestor of rice and maize, followed by subsequent diploidization *en route *to modern maize [49-51].

### Expression divergence between duplicated *ZmARF *genes

The presence of duplicated *ZmARF *genes raises the question about their functional redundancy. According to the evolutionary models, duplicated genes may undergo different selection processes: nonfunctionalization where one copy loses the function, hypofunctionalization where expression/function of one copy decreases, neofunctionalization where one copy gains a novel function, or subfunctionalization where the two copies partition or specialize into distinct functions [52-54]. These evolutionary fates may result in divergence of expression patterns or protein structure. Evidence for divergence between the duplicate genes could be inferred from expression pattern of *ZmARF5 *and *31 *genes. *ZmARF5 *was highly expressed in seed embryos during germination but the transcript of *ZmARF31 *was very low. In addition, possible subfunctionalization shifts the expression pattern trends of gene pairs. For example, the mRNA abundance of *ZmARF2 *peaked in dry mature embryo but *ZmARF17 *was highly expressed in embryos at 24 h after seed imbibition.

### Regulation of *ZmARF *gene expression

Since ARFs are transcription factors that regulate expression of auxin response genes, it would be interesting to determine the response of Zm*ARF *gene to auxin treatment. It has been reported that *Arabidopsis ARF4*, *5*, *16 *and *19 *and rice *OsARF1 *and *23 *transcripts increased slightly in response to auxin, while *OsARF5*, *14 *and *21 *decreased marginally [15,16,55-57]. In the present study, we found that most of the *ZmARF *genes were responsive to exogenous auxin treatment but the auxin responsive elements were only detected in promoter region of 14 *ZmARF *genes. Therefore, the underlying mechanism for auxin inducibility of *ZmARF *genes needs to be elucidated.

There is a growing body of work showing the posttranscriptional regulation of ARF transcript abundance by micro-RNAs (miRNA or miR) and *trans*-acting-small interfering RNAs (ta-siRNA) [42]. Regulation of *AtARF6 *and *8 *by miR167 is important for development of anthers and ovules [39-41,58], regulation of *AtARF17 *by miR160 is important for *Arabidopsis *growth and development [40] and regulation of *AtARF10 *and *16 *by miR160 plays a role in root cap formation [40,56]. The regulation of *AtARF3 *and *4 *targeted by *TAS3 ta-siRNAs *is required for proper leaf development [59] and juvenile to adult phase changes [60-62]. The target sites of these three small RNAs were also detected in maize *ARF *genes, and are widely conserved in dicots and monocots. Furthermore, our transgenic analysis exhibited that increased miR167 level could cause degradation of transcripts for six potential targets (*ZmARF3*, *9*, *16*, *18*, *22 *and *30*), indicating that *ZmARF *genes showed posttranscriptional regulation. However, we found that the expression patterns of *ZmARF *genes which contained the same target sites varied considerably in embryos during seed development and germination, for example the expression of *ZmARF11, 12, 23, 24 and 26 *with two potential *TAS3 ta-siRNAs *recognition sites. Thus, we speculate that some unidentified factors may also play a role in regulating expression of these genes, which could be highly specific to a selected tissue type or developmental program with the possibility that miRNA and tasiRNA may have functions in very discreet regions.

### Potential functions of *ZmARF *genes during seed development

Seed development is considered as a physical link between parents and sporophytic generation in plants [63]. Auxin signaling is thought to play an important role in embryo development. For example, a higher level auxin is detected in root apex and ends of cotyledon primordia from heart to mature embryo in *Arabidopsis *[64]. Defects in *Arabidopsis *embryo patterning was observed in *arf5 *mutants, which enhanced in *arf5arf7 *double mutants [65]. In this study, seven *ZmARF *genes, including *ZmARF1, 10, 13, 14, 18, 22 *and *25*, appeared to be constitutively expressed in developing embryos, whereas the transcripts of other *ZmARF *genes exhibited dynamic expression patterns, suggesting the partitioning of functions between these genes in embryo development.

In most flowering plants, seed germination is the first and may be the foremost growth stage in the plant's life cycle. Genetic evidence supporting a role of *ARF *genes in germination has been obtained from the analysis of regulation of *AtARF10 *by miR160 [46]. It has been reported that transgenic seeds of Arabidopsis expressing a miR160-resistant form of *AtARF10 *(*mARF10*) are hypersensitive to germination inhibition by exogenous ABA, whereas ectopic expression of miR160 results in a reduced sensitivity to ABA [46]. In the present study, Zm*ARF2*, *5*, *8*, *15*, *17 *and *21 *are predicted to be the targets for miR160, which falls in the same subfamily with *AtARF10*, *16 *and *17*. Expression analysis demonstrated that mRNA accumulation of *ZmARF3, 4, 6, 8*, *10*, *12*, *13*, *14*, *15*, *17*, *18*, *19*, *21, 22, 25, 28 *and *30 *gradually increased during seed germination, reaching its peak in embryos after 24 h of seed imbibition and decreased in later stage, whereas *ZmARF2 *showed high transcript accumulation in dry seed embryos. In addition, dynamic expression patterns were also observed for other *ZmARF *genes. Collectively, we speculate that *ZmARF *genes may be involved in diverse aspects of developmental processes during seed germination.

## Conclusions

Maize *ARF *gene family is expanded as compared to *Arabidopsis *and rice reflecting a succession of maize genomic rearrangements and expansions due to extensive duplication and diversification that frequently occurred in the course of evolution. The expressions of maize *ARF *genes are regulated by auxin and small RNAs. Dynamic expression patterns of *ZmARF *genes in embryo at different stages were observed, which suggest that these genes may be involved in seed development and germination.

## Methods

### Maize *ARF *gene identification

A local implementation of NCBI BLASTX was used for sequence searching. All publicly known *ARF *genes from *Arabidopsis *(*AtARF1-AtARF23*) [15] and rice (*OsARF1-OsARF25*) [16] were used in initial protein queries. Maize (*Zea mays*) proprietary ESTs and their assemblies, publicly available ESTs, CDS, GSS, BACs, and The Institute for Genomic Research genomic GSS assemblies AZM_4, AZM_5 http://maize.tigr.org/, and MAGI_4 http://magi.plantgenomics.iastate.edu/ were the source for sequences [33]. All potential hits to conserved regions of *ARF *gene family were assembled and additional rounds of searching were performed to achieve the most possible complete genomic and/or transcript sequences. The Pfam database http://pfam.sanger.ac.uk/search was used to confirm each predicted ZmARF protein sequence as an auxin response factor protein. In addition, full length coding cDNA sequences of 11 *ZmARF *genes (*ZmARF1, 3, 7, 9, 11, 14, 17, 20 22, 24 *and *30*) were further confirmed by RT-PCR amplification, cloning and sequencing.

### Isolation of total RNA and reverse-transcription

Total RNA was isolated using a standard Trizol RNA isolation protocol (Life Technologies, USA) and treated with DNase (Promega Corporation, USA) following the manufacturer's instructions. The amount and quality of the total RNA was confirmed by electrophoresis in 1% formamide agarose gel. For each plant tissue sample, 2 μg of total RNA was reverse transcribed to cDNA in 20μl reaction using M-MLV reverse transcriptase (Promega Corporation, USA). Reverse transcription was performed at 37°C for 60 min with a final denaturation step at 95°C for 5 min.

### RT-PCR amplification, cloning and sequencing

RT-PCR amplification conditions were optimized from the method described from the previous study [66]. The primer information is given in Additional file 1. For gene cloning, PCR amplified samples were separated in 1.0% agarose gel, purified with Sephaglas BandPrep kit (Amersham Pharmacia, USA), cloned into pGEM-T vector (Promega Corporation, USA) and sequenced by an ABI PRISM 3730 capillary sequencer (PE Applied Biosystem, USA) using an ABI Prism Dye Terminator sequencing kit (PE Applied Biosystem, USA) and either vector or sequence specific primers.

### Mapping *ZmARF *genes on maize chromosomes

All the sequenced contigs of B73, representing the 10 maize chromosomes, have been physically constructed and are publically available. The BAC based physical map generated by fingerprinted contigs http://www.genome.arizona.edu/fpc/maize/WebAGCoL/WebFPC/ was used to find the nearest available markers to position *ARF *genes on the genetic IBM2 map http://www.maizegdb.org. The distinctive name for each of the *ZmARFs *identified in this study was given according to its position from the top to the bottom on the maize chromosomes 1 to 10.

### Sequence and phylogenetic analysis of ZmARFs

The exon/intron structures of the *ZmARF *genes were determined from alignments of cDNA and genomic sequences using gene structure displayer http://gsds.cbi.pku.edu.cn/. The identification of small RNA target sites in *ZmARF *genes was performed by using miRanda software http://www.miranda-im.org/. Multiple-sequence alignments of ZmARF proteins were carried out using the Clustal_X (version 1.83) program [67]. The protein sequences of *Arabidopsis *and rice auxin response factors were obtained from the TIGR database and phylogenetic analysis was performed with MRBAYES 3.1.2 program [68] by Bayesian method [69] and the bootstrap test was carried out with 1,000,000 iterations.

### Generation of *Ubi::MIR167b *maize transgenic plants and expression analysis of *ZmARFs*

A 426 bp fragment for the *pre-MIR167b *precursor was amplified from maize inbred line Zong3 with the gene-specific primers (5'-GAGGATTGTTTACGCCACCTT-3' and 5'-GGAGAGAATTGAAAGAGAGAGAGGAG-3'). This DNA fragment was verified by sequencing, and ligated into the plant transformation vector pCAMBIA3300, downstream to the *ubiquitin *promoter. This construct was introduced into maize inbred line Zong3 by *Agrobacterium*-mediated transformation [70] and the transgenic lines were confirmed by PCR primers (5'-GGTGGACGGCGAGGTCGCCG-3' and 5'-TCGGTGACGGGCAGGACCGG-3') specific to *bar *gene.

For expression analysis, roots of Zong3 (wild type) line and three homologous *pre-MIR167b *overexpressing lines (*pUBI::MIR167b-22*, *-36 *and *-47*) were harvested from 8-day-old seedlings grown in a container with tap water under 16 h of light (25°C) and 8 h of dark (20°C). Seedlings were grown in a completely randomized design and three batches of seedlings were used as separate biological replicates. Relative mRNA abundances of six *AtARF6/8*-like *ZmARFs *(*ZmARF3*, *9*, *16*, *18*, *22 *and *30*) were analyzed by real-time RT-PCR.

### Auxin treatment

Seeds of maize inbred line B73 were placed embryo side down on two pieces of Whatman No. 1 filter paper placed in a plastic Petri dish. After overnight imbibition, maize seeds were transferred into a container with tap water and grown in a chamber at constant temperature, (25°C) relative humidity (80%), and subjected to 12 h light/dark cycle (12 h day/12 h night) for 8 days, then transferred to a 5 μM αNAA solution. Control plants were grown in distilled water. Primary roots were isolated after 0, 1, 2 and 3 h of αNAA exposure and from control plants at the same time points and three replicates were harvested for RNA extraction.

### Tissue preparations

For maize inbred line B73, embryos of 15 and 20 days after pollination were harvested from greenhouse-grown plants in a sand/peat under 16 h of light (25°C) and 8 h of dark (20°C), and seed embryos at 0, 12, 24 and 48 h after imbibition in a chamber at 25°C with 12 h light/dark cycle (12 h day/12 h night) were detached from seeds. Eight-day-old seedling leaves and roots were harvested for expression analysis.

### Real-time RT-PCR analysis

Five commonly used housekeeping genes of maize (Additional file 7) were evaluated by geNorm algorithm [71]. Initial steep decrease in average *M *value of each gene is shown in Additional file 8, which firmly demonstrates that *β-Actin *is the most stable control gene (with the lowest *M *value). Real-time RT-PCR reactions were performed according to previous study [72], *β-Actin *was used as an internal control. Details of primers used in this study are given in Additional file 9, and 2μl aliquots of the cDNA were subjected to expression analysis. The reaction conditions were as follows: 94°C for 3 min, followed by 40 cycles at 94°C for 30 s, 55-65°C for 30 s, 72°C for 30 s, and a final extension of 72°C for 5 min. Quantification of results were obtained by CFX96™ Real-Time PCR Detection System (Bio-Rad Laboratories, Inc., USA). cDNAs from three biological samples were used for analysis and all the reactions were run in triplicate. The threshold cycles (Ct) of each tested genes were averaged for triplicate reactions and the values were normalized according to the Ct of the control products of *β-Actin *gene.

## Authors' contributions

HX, GG, GX performed the computational analysis of ARF gene family. RNP coordinated and helped to draft the manuscript. ZH and YZ prepared the materials and did PCR analysis. QS and ZN designed the experiment and prepared the manuscript. All the authors have read and approved the final manuscript.

## Supplementary Material

Additional file 1 **Primer sequences for full-length cDNA cloning of 13 *ZmARFs***. Click here for file

Additional file 2 **Sequence identity matrix of maize ARF proteins**. BioEdit program were employed to examine sequence identity of 31 maize ARF proteins.a Click here for file

Additional file 3 **Sequence alignment maize ARF proteins**. Clustal_X program were employed to examine sequence features of 31 maize ARF domains. Click here for file

Additional file 4 **miR160, 167 and *TAS3 *target site prediction for *ZmARF *genes**. Click here for file

Additional file 5 **Promoter analysis of maize *ARFs***. Auxin response elements are shown in the list. Click here for file

Additional file 6 **The relative expression levels of 31 *ZmARFs *in three tissues of maize inbred line B73**. Leaves (8-day-old seedling), roots (8-day-old seedling) and embryos (15d after pollination) of maize inbred line B73 were used for real-time RT-PCR analysis. Click here for file

Additional file 7 **Primers used for stability evaluation of housekeeping gene expression**. Click here for file

Additional file 8 **Stability evaluation of housekeeping gene expression**. Auxin treated 8-day-old primary roots (A) and embryos during seed development and germination (B) were used for evaluating the stability of five housekeeping genes. Click here for file

Additional file 9 **Primers used for expression study of *ZmARF *genes**. Click here for file
