# Supplementary material for: Genome-wide identification and expression profiling of auxin response factor (ARF) gene family in maize
Source: BMC Genomics. 2011 Apr 7;12:178. doi: 10.1186/1471-2164-12-178 (PMC3082248; doi:10.1186/1471-2164-12-178)
Supplement: Additional file 1 — Primer sequences for full-length cDNA cloning of 13 ZmARFs. [file 1471-2164-12-178-S1.DOC]

**Additional file 1. Primer sequences for full-length cDNA cloning of 13 *ZmARF* genes**

| Genes | Forward primer (5′-3′) | Reverse primer (5′-3′) |
| --- | --- | --- |
| *ZmARF1* | ATGGAGGCGCCGGGGACGAG | CTAGTCGCCGGTGACCCTC |
| *ZmARF3* | TAGAGGTTAGGTTCCGGTGT | TGCGAGCACAGTAGAGCAGG |
| *ZmARF9* | ATGAACCTCTCACCGCCC | TCGGTCTCAGTAGTCCAG |
| *ZmARF10* | ATGCCGCCCGCAACAGCCAT | CCACCAAGAGCAAACCACC |
| *ZmARF12* | TCCGTCCATCCATCGCCTCG | GATATGCCATGTGGATCAGATCA |
| *ZmARF16* | CGGGAGGAGAATGAAGCTCT | TCAGAACTCGACCGAACCCA |
| *ZmARF18* | ATGAGGCTCTCGTCGTCG | TGCTGCTCATCCCAACTC |
| *ZmARF20* | ATGAAGCAGTCCCCGGCCAG | TCACTCAAATTGGTCGTAGA |
| *ZmARF22* | ATGGAGTCTCAGATCCCCAG | GTCATGGTAACTTACTGCC |
| *ZmARF24* | ATGGCGGGGATCGACCTCAAC | AGATCATCATATCCACTG |
| *ZmARF25* | ATGCCGCCCGCAGCCATGG | CCTACTCGTTTGCGGCGGC |
| *ZmARF27* | ATGATGCTTCAGCCTGTGA | TCAGGCCCTCCATGCATT |
| *ZmARF30* | AGGCCTAAGGGTGTGTTTGG | GCCGAAACCTAACCTCCTCT |
