# Supplementary material for: Genome-wide identification and expression profiling of auxin response factor (ARF) gene family in maize
Source: BMC Genomics. 2011 Apr 7;12:178. doi: 10.1186/1471-2164-12-178 (PMC3082248; doi:10.1186/1471-2164-12-178)
Supplement: Additional file 3 — Sequence alignment maize ARF proteins. Clustal_X program were employed to examine sequence features of 31 maize ARF domains. [file 1471-2164-12-178-S3.RTF]

 
                                                                                                  DBD

                    10          20         30          40         50          60          70         80          90        100                  
          ....|....|....|....|....|....|....|....|....|....|....|....|....|....|....|....|....|....|....|....| 
ZmARF1   -----------------MEAPGTSSGG----------AGG-EGAG---GTKVNEELWYACAGPLVALPPAGSLVVYFPQGHSEQV--AASMRKDADAKIP 
ZmARF2   ---------------MKEVAEE--------------------------RCL-DPQLWHACAGGMVQMPPVRSRVYYFPQGHAEHAHGGG-AADLAGARA- 
ZmARF3   ----------------MSSSSAASIGQ----------PPPPAPPPEEEKKCLNSELWHACAGPLVCLPTVGTRVVYFPQGHSEQV--AASTNKEVDGHIP 
ZmARF4   ------MTSSYEKATSGVLRNAAALLD----------EMQLMGETQGAKKVINSELWHACAGPLVCLPQRGSLVYYFPQGHSEQV--AATTKKIPNSRIP 
ZmARF5   --------MGPQPPLHLPAANG--------------------------DSIVDRDVWLACAVPLSRLPAVGAEVYYFPHGHAEQCPA-----HLPAPLP- 
ZmARF6   ---MDAPNPGAAAGPGMPS------------------------------DALYQELWHACAGPLVTVPRQGERVYYFPQGHMEQL--EASAHHQQLDQYL 
ZmARF7   ------MAQGAGRDP----------------------------------EELFEELWRACAGPLVELPQTNERVFYFLQGHLEQL--QEPTDPALLAEQI 
ZmARF8   -----MITFAD-LTEPAAAGAE--------------------------RCV-DRQLWLACAGSMCTVPLVGASVCYFPQGHAEHALGLDGAADLSAARV- 
ZmARF9   ---------------------MSEP----------------SLESDGEQRCLNSELWHACAGPLVSLPVVGSRVVYFPQGHSEQV--AASTNKEVDAQIP 
ZmARF10  --------MAPPSQPPSNSG-----------------------------DPLYPELWRACAGPLVTVPRVGDLVFYFPQGHIEQV--EASMNQVAGNQ-M 
ZmARF11  ----MGIDLNMADGESQERRPP---------------------------PAVCRELWHACAGPVVALPRRGSLVVYLPQGHLAAA--------GGGNVAV 
ZmARF12  ---MAGIDLNTVEEEDEEEAEA---------------LPLPGPGG---RGAVCLELWHACAGPVPPLPRKGSAVVYLPQGHLEHIG----GDAARGAAAS 
ZmARF13  ---MTSSAAAAAAAAQVAGGGGGGEGD----------AAAAARGGGGTEDGMYTELWNLCAGPLVTVPRVGDKVYYFPQGHIEQV--EASTNQVAEQH-M 
ZmARF14  -----MASAGVAGCSGSAG------------------------------DALFRELWHACAGPLVTVPRQGELVYYFPQGHMEQL--EAST-DQQLDQHL 
ZmARF15  -----MITFAD-LTEPAAAGAE--------------------------RCV-DRQLWLACAGGMCTVPPVGASVYYFPQGHAEHALGLAGAADLSAARV- 
ZmARF16  --------------------MPSAQ--------------MFFFPEAEEHKCLNSELWHACAGPLVSLPSVGSRVVYFPQGHGEQV--AASTNKEMEAQIP 
ZmARF17  ---------------MKEAGEE--------------------------RCL-DPQLWHACAGGMVQMPPVRSRVYYFPQGHAEHAHGGGGATDLAGARAR 
ZmARF18  -MR------LSSSSG----SVLPAQ--------------PGSPEAVEEHKCLNSELWHACAGPLVSLPAVGSRVVYFPQGHSEQV--AASTNKEIESQIP 
ZmARF19  --------------MERERESD--------------------------RCL-DPQLWHACAGGMVQMPAVHSKVYYFPQGHAEHAQG---PVDLPAGRV- 
ZmARF20  MKQSPASSGVTAAPVVPAPAPAAASTG----------AAP-PCEGERKAPAINADLWYACAGPLVSLPPVGSLVVYFPQGHSEQV--AASMQKDIDAHVP 
ZmARF21  -----MITFVDSAAMELERESG--------------------------RCLVDPQLWHACAGGMVQMPPVHSRVYYFPQGHAEHAQGHA-HADLPAGRV- 
ZmARF22  -MSPVHSPMISRSIRNLQIAVLSYQNK----------TNTSAVTDVEEHKCLNSELWHACAGPLVSLPAVGSRVVYFPQGHSEQV--AASTNKEMESQIP 
ZmARF23  ----MGIDLNAVG----EDDPA---------------------------GAVCPELWHACAGAGVALPRRGSAVVYLPQAHLAAGG-----CDGGGGLAP 
ZmARF24  ---MAGIDLNDTVEEDEEEAEPGNACSQQSRTSSAATFPPPPPNQPRPSAAVCLELWHACAGPVAPLPRKGSVVVYLPQGHIEHLG----DAAAAGGGAP 
ZmARF25  --------MALPSQAPSNSG-----------------------------DPLYPELWRACAGPLVTVPRVGDLVFYFPQGHIEQV--EASMNQVAGNP-M 
ZmARF26  ----MGIDLNMVDGEGHERRPP---------------------------PVTCRELWHACAGPVVALPRRGSLVVYLPQGHLAAA--------GGGDVAA 
ZmARF27  -------------------MKDHGSGG----------VTPSPAEGE--KKPINSELWHACAGPLVSLPPVGSLVVYFPQGHSEQV--AASMHKELDT-VP 
ZmARF28  ---MVAAAAAAGGGADAGCGGGGG---------------------GGGKDALFVELWKACAGPLSSVPLLGEKVYYFPQGHIEQV--EASTNHLAEHQGT 
ZmARF29  ------MASSQEKATSGVLRNAAALLD----------EMQLMGETQGAKKVINSELWHACAGPLVCLPQRGSLVYYFPQGHSEQV--AATTKKIPNSRIP 
ZmARF30  ----------------MSSSSAASIGQ----------QP-----PEEEKKCLNSELWHACAGPLVCLPTVATRVVYFPQGHSEQV--AASTNKEVDGHIP 
ZmARF31  --------MEPQPPLPLANGHG--------------------------NSIVDRDVWLACAAPLSRLPTVGDDVYYFPDGHAEQCPA-----HLPAPLP- 
            
                                                             DBD


                   110        120        130        140         150        160         170        180         190        200         
          ....|....|....|....|....|....|....|....|....|....|....|....|....|....|....|....|....|....|....|....| 
ZmARF1   SYPNLSSKLICILRSVTMLADPDTDEVYARMTLQPVSNVTHCDKET------------LLATELALKQ-TRPQTEFFCKTLTASDTSTHGGFSVPRRAAE 
ZmARF2   ----LPSLVLCSVTGVRFLADPETDEVFAKIRLVPVAPGEVEFREP-------------DEFSVDPADAR-EKLSSFAKTLTQSDANNGGGFSVPRYCAE 
ZmARF3   NYPNLPPQLICQLHDVTMHADVETDEVYAQMTLQPLN--PQEQNDA------------YLPAEMGIM--SKQPTNYFCKTLTASDTSTHGGFSVPRRAAE 
ZmARF4   NYPSLPSQLLCQVHNITLHADKETDEIYAQMTLQPVH----SETDV------------FPIPTLGAYTKSKHPSEYFCKNLTASDTSTHGGFSVPRRAAE 
ZmARF5   ----APHLFPCTVAGVSLGADDETNEVFAKISLSPGPHRGPAAAC--------------RTDP-TSDCPPQE-LSYFTKELTQSDANNGGGFSVPRYCAD 
ZmARF6   PMFDLPPKILCRVVNVELRAEADSDEVYAQIMLQPEA----DQNE-------------LTSLDAEPQEREKCTAHSFCKTLTASDTSTHGGFSVLRRHAE 
ZmARF7   KMFQVPNKILCKVVNVELKAETETDEMYAQITLQPEP----DQVD------------LPQLPEPPLQETSRPVVHSFCKILTPSDTSTHGGFSVLRRHAN 
ZmARF8   -----PALVPCRVTAVRYMADPDTDEVFARIRLVPLRGG--EAHAG-------------GLDDDVAAADEQEKPASFAKTLTQSDANNGGGFSVPRYCAE 
ZmARF9   NYPNLPPQLICQLHNVTMHADAETGEVYAQMTLQPLS--PEEQKEP------------FLPIELGAG--SNQPTNYFCKTLTASDTSTHGGFSVPRRAAE 
ZmARF10  RLYDLPPKLLCRVLNVELKAETDTDEVYAQIMLMPEP----EQTD-----------VPAEKPSSAPAASPRPAVRSFCKTLTASDTSTHGGFSVLRRHAD 
ZmARF11  D---LPPHVACRVADVELCADAATDEVYARLALVAEGE-AFGRNLRGGGVDGDDDMEDFDVER------KSRMLHMFCKTLTASDTSTHGGFSVPRRAAE 
ZmARF12  ---AVPPHVLCRVVDVTLHADGATDEVYARVSLLPEDE-DAEKRAQAQARVREDEDRRDGEDGGAMRP-LARTPHMFCKTLTASDTSTHGGFSVPRRAAE 
ZmARF13  QFYDLPWKILCEVMNVELKAEPDNDEVYAQLTLLPES----KQPE--------ENGSSEEMPASPPAALARPRVHSFCKTLTASDTSTHGGFSVLRRHAD 
ZmARF14  PLFDLPPKILCKVVNVELRAETDSDEVYAQIMLQPEA----DQSE-------------PTSPDSEPPEPERCNVYSFCKTLTASDTSTHGGFSVLRRHAE 
ZmARF15  -----PALVPCRVTAVRYMADPDTDEVFARIRLVPLRGGDADADAG-------------GVEDDAAAADEQEKPASFAKTLTQSDANNGGGFSVPRYCAE 
ZmARF16  NYPSLPPQLICQLHNVTMHADAETDEVYAQMTLQPLS--PQELKDP------------FLPAELGTA--SNQPTNYFCKTLTASDTSTHGGFSVPRRAAE 
ZmARF17  P---LPPLVLCTVAGVRFLADPETDEVFAKIRLVPAAPGEVEFGEP-------------REFGIDPEDAR-EKLSSFAKTLTQSDANNGGGFSVPRYCAE 
ZmARF18  NYPNLPPQLICQLHNVTMHADAETDEVYAQMTLQPLN--PQELKDP------------YLPAELGSA--NKQPTNYFCKTLTASDTSTHGGFSVPRRAAE 
ZmARF19  -----PALVLCRVAAVRFMADPDTDEVFAKIRLAPVRPNEPGYADD-------------AIGAAAASGAQEDKPASFAKTLTQSDANNGGGFSVPRYCAE 
ZmARF20  SYPNLPSKLICLLHSVTLHADPDTDEVYAQMTLQPVN---TYGKEA------------LQLSELALKH-ARPQMEFFCKTLTASDTSTHGGFSVPRRAAE 
ZmARF21  -----PALVLCRVDAVRFLADPDTDEVLARVRLAPVRPNEPDHAD------------------AAAPGAREDKPASFAKTLTQSDANNGGGFSVPRYCAE 
ZmARF22  SYPNLPPQLICQLHNVTMQADAETEEVYAQMTLQPLN--PQELKDP------------YLPAELGLV--SKQPTNYFCKTLTASDTSTHGGFSVPRRAAE 
ZmARF23  APPRVPPHVVCRVVDVELRADAATDEVYARLALVAMDT-MFGRNINDGETEEKNGEEEDGDGE------KKHASHMFCKTLTASDTSTHGGFSVPRRAAE 
ZmARF24  PPVALPPHVFCRVVDVTLHADASTDEVYAQLALVAENE-DVARRLRGRS---EDGSAEDGDEGETVKQRFSRMPHMFCKTLTASDTSTHGGFSVPRRAAE 
ZmARF25  RLYDLPSKLLCRVLNVELKAETDTDEVYAQIMLMPEP----EQND-----------VAAEKTSSGSAAPPRPAVRSFCKTLTASDTSTHGGFSVLRRHAD 
ZmARF26  D---LPPHVVCRVADVELCADAATDEVCARLALVAEGE-AFGRNLGGGGVEGDDGMEDFDAER------KSGMLHMFCKTLTASDTSTHGGFSVPRRAAE 
ZmARF27  SYPSLPSKLICKLLSLTLHADSETDEVYAQMMLQPVN---KYDRDA------------MLASELGLKQ-NKQPTEFFCKTLTASDTSTHGGFSVPRRAAE 
ZmARF28  PLYNLPWKIPCKLMNMELKAEPDTDEVYAQLTLLPDK----KQDENTSARVENEEAEEEVVPHVPPATSEGLRIHSFCKTLTASDTSTHGGFSVLRRHAD 
ZmARF29  NYPSLPPQLLCQVHNITLHADKETDEIYCQMTLQPLH----SETDV------------FPIPTLGAYTKSKHPTEYFCKNLTASDTSTHGGFSVPRRAAE 
ZmARF30  NYPNLPPQLICQLHDVTMHADVETDEVYAQMTLQPLN--PQEQNDP------------YLPAEMGIM--SKQPTNYFCKTLTASDTSTHGGFSVPRRAAE 
ZmARF31  ----APHFFPCTVTDISLGADDKTDEVFAKISLRPG----LAAAS--------------RPDPGSSNSPPREPLSYSIKELSQSDANGGGSFCVPRYCGD 


DBD


                    210        220        230        240         250        260         270        280        290         300         
          ....|....|....|....|....|....|....|....|....|....|....|....|....|....|....|....|....|....|....|....| 
ZmARF1   RIFPHLDFSVQPPAQELQARDLHDAIWTFRHIYRGQPKRHLLTTGWSLFVSGKRLLAGDSVLFIRDG-RQQLLLGIRRANRQPVNLSSSVLSSDSMHI-- 
ZmARF2   TIFPKLDYRADPPVQTVLAKDVHGEVWKFRHIYRGTPRRHLLTTGWSTFVNQKKLVAGDSIVFLRTE-HGELCVGIRRVKRVSCGGMECMSGWNAP---- 
ZmARF3   RVFPPLDFTQQPPAQELIARDIHDVEWKFRHIFRG-PKRHLLTTGWSVFVSAKRLVAGDSVLFIWNE-KNQLLLGIRHATRPQTVMPSSVLSSDSMHI-- 
ZmARF4   KLFPQLDYSMQPPNQELIVRDLHDNMWTFRHIYRGQPKRHLLTTGWSLFVGAKRLKAGDSVLFIRDE-KSQLLVGVRRATRQQPALSSSVLSTDSMHI-- 
ZmARF5   HIFPTLDFDANPPVQKLFMRDTRGNPWQFRHIYRGTPRRHLLTTGWSRFVNAKLLVAGDIVVFMRRH-NGDLIVGLRRTPRYPLVFPRVGSG-AGVDP-- 
ZmARF6   ECLPQLDMSQNPPCQELVAKDLHGTEWHFRHIFRGQPKRHLLTTGWSVFVSSKRLVSGDAFIFMRGE-NGELRVGVRRLMRQVNSMPSSVISSHSMHL-- 
ZmARF7   ECLPALDMSMPTPTQELITKDLHGSEWRFKHIYRGQPRRHLLTTGWSTFVTSKKLIAGDAFVYLRSE-TGQQRVGVRRLVQKQSTMPASVISSQSMHL-- 
ZmARF8   TIFPRLDYAADPPVQNVVAKDVHGTAWKFRHIYRGTPRRHLLTTGWSTFVNQKKLIAGDSIVFLRGD-SGDLHVGIRRAKRGFCGAGGGGGDEAPTP--- 
ZmARF9   KVFPPLDFSQHPPVQELVARDLHDNEWKFRHIFRGQPKRHLLTTGWSVFVSAKRLVAGDSIIFIWND-NNQLLLGIRRANRPQTVMPSSVLSSDSMHI-- 
ZmARF10  ECLPPLDMTQSPPTQELVAKDLHGMEWRFRHIFRGQPRRHLLQSGWSVFVSSKRLVAGDAFIFLRGE-NGELRVGVRRAMRQLSNVPSSVISSQSMHL-- 
ZmARF11  DCFPPLDYNQLRPSQELVAKDLHGAKWKFRHIYRGQPRRHLLTTGWSSFVNKKKLVSGDAVLFLRGD-DGELRLGVRRAIQLKNEALFDDFSCDSTKR-- 
ZmARF12  DCFPPLDYSQQRPSQELVAKDLHGTEWKFRHIYRGQPRRHLLTTGWSAFVNRKKLISGDAVLFLRGE-DGVLRLGVRRAAQLKIVTPIPAPHNQCSSN-- 
ZmARF13  ECLPPLDMTRQPPTQELVAKDLHGVEWRFRHIFRGQPRRHLLQSGWSVFVSAKRLVAGDAFIFLRGD-SGELRVGVRRAMRQQANVPSSVISSHSMHL-- 
ZmARF14  ECLPQLDMTQNPPWQELLAKDLHGNEWHFRHIFRGQPRRHLLTTGWSVFVSSKRLVAGDAFIFLRGE-NGELRVGVRRLMRQLNNMPSSVISSHNMHL-- 
ZmARF15  TIFPRLDYAADPPVQTVVAKDVHGAAWKFRHIYRGTPRRHLLTTGWSTFVNQKKLVAGDSIVFLRGD-SGDLHVGIRRAKRGFCGAGGGGGDDSPAA--- 
ZmARF16  KVFPPLDFNQQPPAQELIAKDLHGNDWKFRHIFRGQPKRHLLTTGWSVFVSAKRLVAGDSVLFIWND-NNQLLLGIRRANRPQTVMPSSVLSSDSMHI-- 
ZmARF17  TIFPKLDYRADPPVQTVLAKDVHGEVWKFRHIFRGTPRRHLLTTGWSAFVNQKKLVAGDSIVFLRTE-HGELCVGIRRAKRVSCGGMECISGWNAP---- 
ZmARF18  KVFPPLDFTQQPPCQELMAKDLHGNEWKFRHIFRGQPKRHLLTTGWSVFVSAKRLVAGDSVLFIWND-NNQLLLGIRRANRPQTVMPSSVLSSDSMHI-- 
ZmARF20  TIFPRLDYSADPPVQTVLAKDVHGVVWKFRHIYRGTPRRHLLTTGWSTFVNQKKLVAGDSIVFMRTE-NGDLCVGIRRAKKGGIGGPEFMHHHHQQPPPP 
ZmARF19  KILPPLDFGMQPPAQELQARDIHDNVWTFRHIFRGQPKRHLLTTGWSLFVGGKRLFAGDSVIFVRDE-RQQLLLGIRRASRQPTNISSSVLSSDSMHI-- 
ZmARF21  TIFPRLDYSADPPVQTVLAKDVHGVVWKFRHIYRGTPRRHLLTTGWSAFVNQKRLVAGDSIVFMRTGGTGDLCVGIRRAKKGGIGGGPEFPHHQPPDGGG 
ZmARF22  KVFPPLDFTQQPPCQELMATDLHGNEWKFRHIFRGQPKRHLLTTGWSVFVSAKRLVAGDSVLFIWND-NNQLLLGIRRANRPQTVMPSSVLSSDSMHI-- 
ZmARF23  DCFPPLDYEQLRPSQELIAKDLHGMKWRFRHIYRGQPRRHLLTTGWSSFINKKKLVSGDAVLFLRGN-DGELRLGVRRAVQLKNEALLEAVNCTDSKL-- 
ZmARF24  DCFPPLDYSQQRPSQELVAKDLHGTEWRFRHIYRGQPRRHLLTTGWSAFVNKKKLVSGDAVLFLRGD-NGELRLGVRRAAQLKNGSAFPALYNQCLNL-- 
ZmARF25  ECLPALDMSQSPPTQELVAKDLHGMEWRFRHIFRGQPRRHLLQSGWSVFVSSKRLVAGDAFIFLRGE-NGELRVGVRRAMRQLSNVPSSVISSQSMHL-- 
ZmARF26  DCFPPLDYNQLRPSQELVAKDLHGAKWKFRHIYRGQPRRHLLTTGWSSFVNKKKLVSGDAVLFLRGD-DGELRLGVRRAIQLKNEALFEDFNSDSTKR-- 
ZmARF27  KIFPPLDFTMQPPAQELIAKDLHDISWKFRHIYRGQPKRHLLTTGWSVFVSTKRLLAGDSVLFIRDE-KSQLLLGIRRASRPQPALSSSVLSSDSMHI-- 
ZmARF28  ECLPPLDMSQHPPNQELVAQDLHGVEWRFRHIFRGQPRRHLLQSGWSVFVSAKRLVAGDAFIFLRGE-NGELRVGVRRALRHQTTIPSSVISSHSMHL-- 
ZmARF29  KLFPQLDYSMQPPNQELIVRDLHDNMWTFRHIYRGQPKRHLLTTGWSLFVGAKRLKAGDSVLFIRDE-KSQLLVGVRRATRQQPALSSSVLSTDSMHI-- 
ZmARF30  RVFPPLDFTQQPPVQELIARDIHDVEWKFRHIFRGQPKRHLLTTGWSVFVSAKRLVAGDSVLFIWNE-KNQLLLGIRRASRPQTVMPSSVLSSDSMHI-- 
ZmARF31  HVWPKVDFEADPPMQNLVMHDTTGKQWEFRHVYRAKQPRHVLTTGWSKFVNAKLLVAGDIIVFMRRP-NGDLIVGLRRMPRYAGTLHRPGTGGDAQDP-- 

DBD
 
 
                   310         320        330         340        350        360         370        380        390         400         
          ....|....|....|....|....|....|....|....|....|....|....|....|....|....|....|....|....|....|....|....| 
ZmARF1   --------------------------------------GILAAAAHAAANNSQFTVFYNPR--ASPSEFVIPFAKYQKAVYSNQLSLGMRFRMMFETEES 
ZmARF2   ---GYGALSAFLKDEEGKMMKSHGGYMRG---RGKVKITDVVNAASLAASGQPFEVVYYPR--ASTPEFVVKAASVQNAMRN-QWCPGMRFKMAF-ETED 
ZmARF3   --------------------------------------GLLAAAAHAAATNSRFTIFYNPR--ASPTEFVIPLSKYIKAVFHTRISVGMRFRMLFETEES 
ZmARF4   --------------------------------------GVLAAAAHAASSGGSFTVYYNPR--TSPSPFVIPLARYNMATYL-QPSVGMRFAMMFETEES 
ZmARF5   ----------------DQP---PPRNARA-----RVPPQDVIEAARLAAEGRSFAVTYFPR--QAAGEFIVPRDEVEGVLAT-RWEPGAQVRMQVMEAED 
ZmARF6   --------------------------------------GVLATASHAISTGTLFSVFYKPR--TSRSDFIVSVNKYLEAKKQ-KISVGMRFKMRFEGDDA 
ZmARF7   --------------------------------------GVLASASHAIRTHSIFLVYYRPR--LSQSQYIVRLNKYLESSKI-GFDVGMRFKMSFEGDDV 
ZmARF8   ---GWHHYAGLIRG---NVSPCAAAKARG-----KVRPEDVAEAARLAAAGQSFEVVYYPR--ASTPEFCVRAAAVRAAMRV-QWSPGMRFKMAF-ETED 
ZmARF9   --------------------------------------GLLAAAAHAAATNSRFTIFYNPR--ASPSEFVIPLAKYVKAVYHTRVSVGMRFRMLFETEES 
ZmARF10  --------------------------------------GVLATAWHAINTKSMFTVYYKPR--TSPSEFIIPYDQYMESVKN-NYSIGMRFRMRFEGEEA 
ZmARF11  --------------------------------------HTLLAVADSLKHKSVFHISYNPR--ATASEYIIPHHKFLKSLNL-PFCIGARINLQCHNEDV 
ZmARF12  --------------------------------------SNLGNVAQAVATKTVFHIYYNPR--LTQSEFIVPYWKFTRSFNQ-PISVGMRCRMRYESDDA 
ZmARF13  --------------------------------------GVLATAWHAVNTGTMFTVYYKPR--TSPAEFVVPCDRYMESLKR-NYPIGMRFKMRFEGEEA 
ZmARF14  --------------------------------------GVLATASHAISTGTLFSVFYKPR--TSRSEFVVSVNKYLEAKNH-KVSVGMRFKMRFEGDES 
ZmARF15  ---GWDHYAGLMRG---NVSPCAAAKARG-----KVRPEDVAEAARLAAAGQSFEVVYYPR--ASTPEFCVRAAAVRVAMRV-QWSPGMRFKMAF-ETED 
ZmARF16  --------------------------------------GLLAAAAHAASTNSRFTIFYNPR--ASPCEFVIPMAKYVKAVYHTRISVGMRFRMLFETEES 
ZmARF17  ---VYGALSAFLKDEEGKITKGPGGYMRG---RGKVEITDVVEAASLAASGQPFEVVYYPR--ASTPEFVVKAASVQNAMRN-QWCPGMRFKMAF-ETED 
ZmARF18  --------------------------------------GLLAAAAHAASTNSRFTIFYNPR--ASPSEFVIPLVKYVKAVYHTRISVGMRFRMLFETEES 
ZmARF19  QGGGYAGFSMFLRGEEDGGKMMAAAATRGKAARVRVRPEEVVEAANLAVSGQPFEVVYYPR--ASTPEFCVKAGAVRAAMRT-QWCPGMRFKMAF-ETED 
ZmARF20  --------------------------------------GVLAAAAHAAANNSPFTIFYNPRRVASPTEFVIPFAKFQKALYSNQISLGMRFRMMFETEEL 
ZmARF21  YGYGYAGFSTFLRGEEDDEGQGQGAGAAGGGRRGRQP-----RGERAAVRGG----LYLPK--ANTQSCASRRGRS-AHHVT-QWCAGMRFKMAF-ETED 
ZmARF22  --------------------------------------GLLAAAAHAASTNSRFTIFYNPR--ASPSEFVIPLAKYVKAVYHTRISVGMRFRMLFETEES 
ZmARF23  --------------------------------------LMLSAVANSLDNRSIFHICFNPR--VGASEFIVPYCKFLKSLNY-HFSVGTRFKVGCENEDA 
ZmARF24  --------------------------------------GSLPNVAHAVATKSVFHIYYNPR--LSQSEFIIPFSKFIKSFSQ-PFSAGSRFKVKYESDDA 
ZmARF25  --------------------------------------GVLATAWHAINTKSMFTVYYKPR--TSPSEFIIPYDQYMESVKN-NYSIGMRFRMRFEGEEA 
ZmARF26  --------------------------------------HSLTAVADSLKNRSVFHISYNPR--ATASEYIIPYAKFLKSLNH-PVCIGARINFQCHNEDV 
ZmARF27  --------------------------------------GILAAAAHAAANSSPFTIFYNPR--ASPSEFVIPLAKYNKALYT-QVSLGMRFRMLFETEDS 
ZmARF28  --------------------------------------GVLATAWHAVNTGSMFTVYYKPR--TSPAEFVVSRARYCESLKR-NYSIGMRFRMRFEGEEA 
ZmARF29  --------------------------------------GVLAAAAHAASSGGSFTIYYNPR--TSPSPFVIPLARYNKATYL-QPSVGMRFAMMFETEES 
ZmARF30  --------------------------------------GLLAAAAHAAATNSRFTIFFNPR--ASPSEFVIPLSKYIKAVFHTRISVGMRFRMLFETEES 
ZmARF31  ----------------DQPPPPPPRNALA-----RVPPKDVMEAARLAAEGRPFTVTYYPR--KAAGEFVVPRNEVEGVLDT-LWEPGSHVLMQFAEAED 


DBD


                   410         420        430         440        450        460         470        480         490        500         
          ....|....|....|....|....|....|....|....|....|....|....|....|....|....|....|....|....|....|....|....| 
ZmARF1   ATR--RYMGTITGISDMDPL---RWKNSQWRNIQVAWDEAAPSERRTRVSLWEVEPVIAPFFIYPSPL-FTAKRPRQPGVTDDDSSEMDNLFK-RTMPWF 
ZmARF2   SSRISWFMGTIASAQVADPI---RWPNSPWRLLQVAWDEPDLLQNVKCVNPWLVEIVSSIPPIHLGP---FSPP-RKKLRVPHHPDFPFDGQLLNPIFHG 
ZmARF3   SVR--RYMGTITEVSDADPV---RWPSSYWRSVKVGWDESTAGERPPRVSLWEIEPLTT-FPMYPSLFPLRVKHPWYSGLAALHDDS-------NALMWL 
ZmARF4   SKR--RCTGTIVGISDYEPM---RWPNSKWRNLQVEWDEHGYGERPERVSLWDIETPEN--MVFSSPLNSKRQCLPSYGVSGLHV----------SSISK 
ZmARF5   TRRTVWADGHVKSLHQN-----------IWRALEIDWD------DSSPLSPNLSRFVN-----------------AWQVELVTHPPLPNGARICNPIASL 
ZmARF6   PER--RFSGTIIGIGSLPAMSKSLWADSDWRSLKVQWDEPSSILRPDRISPWEVEPLDAANP---QSPQPPLRAKRPRPPASPCMVS------------- 
ZmARF7   PIK--KFSGTVVDKGDLSP----QWQGSEWKTLKVKWDEATNLNGPERVSSWEIEPFDASAPAITMPVQPSMKNKRPRETAEGLDIHALEPAQEFWLSGR 
ZmARF8   SSRISWFMGTVAGVQVTDPI---RWPQSPWRLLQVTWDEPDLLQNVKRVSPWLVELVSSMPAIHLAS---FSPP-RKKPRIPAYPEFPFEGQLLNPAFPP 
ZmARF9   SVR--RYMGTITCISDLDSE---RWPNSHWRSVKVGWDESTAGDKQPRVSLWEIEPLMA-FPMYPTAFPLRLKRPWASGLPMFNGGRSDEFARYSSLMWL 
ZmARF10  PEQ--RFTGTIVGCENLDP----LWPDSSWRYLKVRWDEPSTIPRPDRVSPWKIEPASSPPV---NPLVHSSRAKRPRQNVPPPSLESSVLTKEGATKVE 
ZmARF11  SER--RS-GMVVHVSEIDPMK---WPGSKWRSLLVRWEDGVECNGQDRVSPWEIEIAGGSVSVAHSVSASSSKRTKLC-PQGNLDVPTMYVTGNGNGCTD 
ZmARF12  SER--RCTGIIIGSREADPI----WYGSKWKCLVVRWDDGIECRWPNRVSPWEIELTG-SVSGSQ-MCAPSSKRLKPCLPQVNPEIVLPN--GSVSSDFA 
ZmARF13  PEQ--RFTGTIVGNVDPDQA---GWAESKWRYLKVRWDEASSIPRPERVSPWQIEPAVSPPP---INPLPVHRPKRPRSNAVASLPESSAPTKEAAPKVT 
ZmARF14  PER--RLSGTIIGLGSMPANSTSPWANSDWRSLRVQWDEPSAILRPDRVSPWELEPLDATNP---QPPQPHLRNKRARPPALLSIAP------------- 
ZmARF15  SSRISWFMGTVAGVQVTDPI---RWPQSPWRLLQVTWDEPDLLQNVKRVSPWLVELVSSMPAIHLAS---FSPP-RKKPRIPAYPEFPFEGQLLNPAFPP 
ZmARF16  SVR--RYMGTITGISDLDPV---RWPNSHWRSVKVGWDESTAGEKQPRVSLWEIEPLTT-FPMYPSPFALGLKRPWPAGLPSLYGGRGDGLT--SSLMWL 
ZmARF17  SSRISWFMGTIASAQVADTI---RWPNSPWRLLQVSWDEPDLLQNVKCVNPWLVEIVSSIPPIHLGT---FSPP-RKKLRVAQHPDFPFEGQLLNPIFHG 
ZmARF18  SVR--RYMGTITGICDLDSV---RWPNSHWRSVKVGWDESTAGERQPRVSLWEIEPLTT-FPMYPSPFPLRLKRPWPTGLPSLHGGKDDDLA--NSLMWL 
ZmARF19  SSRISWFMGTVSAVHVADPI---RWPNSPWRLLQVAWDEPDLLQNVKRVSPWLVELVSNMPVIHHLTATPFSPP-RKKLCVPLYPELPLEGQFPAPMFHG 
ZmARF20  GMR--RYMGTITGITDLDPV---RWKNSQWRNLQVGWDESAAGERRNRVSMWEIEPIAAPFFICPQPF-FGVKRPRQ--IDDESSEMENLFK--RAMPWL 
ZmARF21  SSRISWFMGTVAAVQVADPI---RWPNSPWRLLQVAWDEPDLLQNVKRVSPWLVELVSSTPAIHHLT--PFSPPSRKKLCIPLYPEG---HQLPAPMFHG 
ZmARF22  SVR--RYMGTITGISDLDSV---RWPNSHWRSVKVGWDESTAGDRQPRVSLWEIEPLTT-FPTYTSPFPLRLKRPWPTGLPSLHGGKDDDLA--NSLMWL 
ZmARF23  NER--SF-GLIIGISEVDPIH---WPGSKWKSLLIKWDGATKYSHQNRVSPWDIEGVGSSVSVTHRLSSSVSKRTKLCFPPSDLDTPILD--GNGRPDSV 
ZmARF24  SER--RCTGIIAGIGDADPM----WRGSKWKCLMVRWDDDVDFRQPNRISPWEIELTS-SVSGSH-MSAPNAKRLKPCLPHVNPDYLVPN--GSGRPDFA 
ZmARF25  PEQ--RFTGTIVGCENLDP----LWPDSSWRYLKVRWDEPSTIPRPDKVSPWKIEPASSPPV---NPLPLS-RGKRPRQNAPPPSPESSVLTKEGATKID 
ZmARF26  SER--RS-GVVVRISEIDPMK---WPGSKWRSLLVRWEDGAECNGQDRVSPWEIEIAGGSVSVSHSLSASSSKRTKLC-PQGNLDVPAMYVTGNGCTDSM 
ZmARF27  GVR--RYMGTITGIGDLDPL---RWKNSHWRNLQVGWDESTASERRTRVSIWEIEPVATPFYICPPPF-FRPKLPKQAGMPDDENEVESAFK--RAMPWL 
ZmARF28  AEQ--RFTGTIVGICVSDPS---GWADSKWRSLKVRWDEASSVPRPERVSPWQIEPAVSPSP---VNPLPVR-FKRSRSSVNASPSDVSTVNREVASKVM 
ZmARF29  SKR--RCTGAIVGISDYDPM---RWPNSKWRNLQVEWDEHGYGERPERVSIWDIETPEN--MVFSSPLNSKRQCLPSYGVPGLQIGSVN-----MSSIPR 
ZmARF30  SVR--RYMGTITEVSDADPV---RWPSSYWRSVKVGWDESTAGERPPRVSLWEIEPLTT-FPMYPSLFPLRVKHPWYSGVAALHDDS-------NALMWL 
ZmARF31  TRRTMWADGHVKAIHQK-----------IWRALEIDWDV-----ASSAISAQLGRFVN-----------------AWQVQRIAYP------SICGGLGDL 


                    510        520        530         540        550        560         570        580         590        600         
          ....|....|....|....|....|....|....|....|....|....|....|....|....|....|....|....|....|....|....|....| 
ZmARF1   GEEVGKRDLSTQNGLVPGLSLVQWMNMQHN-PSLANTVMQPELLNSLAGKPVQTLAAADLSRQISFQPQFLQQNNIQFNTSLLPPQNQQTEQLANVIATP 
ZmARF2   NP-LGPSNGGG-------------ALRCFSDI--APAGIQGARHAQFGLPLTDRQ-LNKLHLGLFQ---------------------------------- 
ZmARF3   RGVTGEGGFHSLNFQSPGIGSWGQQRLHPS-----LLSNDHDQYQAVVAAAAASQSGGYLKQQFLHLQQPMQ---------------------------- 
ZmARF4   PQGSPFGNLQHMPGISSDIALLLLNQSAQN--LGSSIACQQSSFSSIIQNAKQSYFPPTTLGASTG---------------------------------- 
ZmARF5   CHGDVSYPLIG----------------SEIQGPPIPASMQGARHTGPCAAPSESS--------------------------------------------- 
ZmARF6   -------------------ELPSGFG----------------LWKSPIESSCTLSFSEPQRARELFPSI------------------------------- 
ZmARF7   PEQHEKTSVS-----SSEPKHQVAWTSERA----GYSAMSSSICQNSAVTGSWFKGFNSSGSHPSLPEIS------------------------------ 
ZmARF8   SPLPHGQQHHHNFLHAHP-----PSFFPFPDG-SAPAAIQGARHAQFVPSLSDLH-LIHLQSSLLY---------------------------------- 
ZmARF9   RD-GNRG-AQSLNFQGLGASPWLQPRIDYP-----LLGLKLDTYQQMAAAALEEIRAGDHLKQIS-SLLPVQ---------------------------- 
ZmARF10  IDSAQTQHQNSVLQGQEQMTLRNNLTESAD----SDSTVQKPMMWSPSLNGKAHTHFQQRPAMDNWMPLGRRE--------------------------- 
ZmARF11  SVETGKFPRVLQGQDLMSFRTRHVPSAPQT--------VEAAKLQSSGASRFLSNARGCALGGPTSR--------------------------------- 
ZmARF12  GSARFHKVLQGQELLGFKTHDGPAISASQA--------TEARNLQYSDERSNNN------LGIPRLGVR------------------------------- 
ZmARF13  LETQQHALQR-PLQTQDNVAPKSVFGDNSE----LDSAHKSSLRPSGFDLDKSTIGMQRKLGSDIWMRMNRPDG-------------------------- 
ZmARF14  -------------------ELPQVFG----------------FLKSPAEPAQAFSFSRPQQTQELYHSN------------------------------- 
ZmARF15  NPLPHGQHHHH-FLHSHSHQHHHPPFFPFPAG-SAPAAIQGARHAQFVPPFSELH-LTHLQPSLLY---------------------------------- 
ZmARF16  RDRANPG-FQSLNFSGLGMSPWMQPRLDNS-----LLGLQSDMYQTIAAAA----ALQSTTKQVPPSAMQFQ---------------------------- 
ZmARF17  NP-LGPSNS---------------PLRCFSDI--APAGIQGARHAQFGLPLTDYQ-LNQLHLGFN----------------------------------- 
ZmARF18  RDTANPG-FQSLNFGGLGMSPWMQPRLDAT-----LLGLQPDMYQAMATA-----AFQDATKQASPTMLQFQ---------------------------- 
ZmARF19  SPLLGRGGAGG-------------PMCYFPDG--TPAGIQGARHAQFGISLSDLH-LNKLQPGLSP---------------------------------- 
ZmARF20  GEEICIKDAQTHNTTMPGLSLVQWMNMNRPQSSTLNTGIQSEYLRSLSNPAMQNLGAAELARQLYVQNHLLQQNSVQLNASKLPQQVQPINELAKGSLSC 
ZmARF21  SPLVGRG--VG-------------PMRYFPDGGTPPAGIQGARHAQFGISLPDLHHLTRLQSSLSPH--------------------------------- 
ZmARF22  RDTTNPG-FQSLNFGGLGMNSWMQPRLDTS-----LLGLQPDMYQAMATG-----AFQDPTKQASPTMLQFQ---------------------------- 
ZmARF23  ETERFHRVLQGQELVHS-SIHGT--ACSHS--------SDSPRCQGSYGRRFSADAWNCKMNDVMSGP-------------------------------- 
ZmARF24  ESAQFHKVLQGQELLGYRTHDNAAVATSQP--------CEATNMQYIDERSCSNDASNIIPGVPRIGVR------------------------------- 
ZmARF25  TDSAQTPHQNSVLQSQEQMSFRNNLTESTD----SDSTVQKQMMWSPSPNGKVHTNFQPRPAMDNWMPLGRRE--------------------------- 
ZmARF26  ETGKLPRVLQGQELMSFRTCHVP--CAPQT--------AEAAKLQSSDASRFLSNAHGCALGGPTSR--------------------------------- 
ZmARF27  ADDFALKDVQ--NALFPGLSLVQWMAMQQN-PQMLATASPAVQSQYLTSNALGMQDGIGSVSEDPTKRLTMQAQNIGL------PNLQAGSKVDHPAITP 
ZmARF28  VESQQNNLPR-ALHNQGRTQLTGRYRDSTD----VKTAQDLTMWSSGTEQQRNNIAAQTKRCLEGWTQSRTPEG-------------------------- 
ZmARF29  AQGNPIGNLQHMPGMGSELALLLLNQSGQN--IGSPIACQQSSFSSIIQNAKHSYFPPKTFGASTASMKQESMP-------------------------- 
ZmARF30  RGVAGEGGFQSLNFQSPGVGSWGQQRLHPS-----LLSNDHDQYQAVVAAAAASQSDGYLKQQFLHLQQPMQ---------------------------- 
ZmARF31  CY---PLPLME----------------SESQ--------------------------------------------------------------------- 


                   610         620        630         640        650         660       670         680         690        700         
          ....|....|....|....|....|....|....|....|....|....|....|....|....|....|....|....|....|....|....|....| 
ZmARF1   NQLGSVIVPQKAVQNSSSEQKQN-LVTQPLQVSQPMVSVAQPQLVHT-QLQQPQVVLQAQPQ--QPQVIVQAQLQQQQPLVQNH-TTIQSGLQQIQLLQQ 
ZmARF2   ---------------------------------------------------------------------------------------------------- 
ZmARF3   ---------------------------------SPQEQCN-LNPLLQQQILQQASQQQMVSSDAQNIQSVLNPG-------------------------- 
ZmARF4   -------------------------------WNESQQQLNALGIQKGDQVSCDVQPGIDSITAPEMNVKPRVPRSTDSYSSQSISDPNSKSDPKTKTRRS 
ZmARF5   ---------------------------------------------------------------------------------------------------- 
ZmARF6   ---------------------------------------------------------------------------------------------------- 
ZmARF7   ---------------------------------------------------------------------------------------------------- 
ZmARF8   ---------------------------------------------------------------------------------------------------- 
ZmARF9   ---------------------------------QPQNLSGGLDPLYGNPVLQQMQFQSQQ-SSLQVVQQGYGPNASDSGFLQN--------------QLQ 
ZmARF10  ----------------------------------------NDFKDTRSAFKDARTASQSFGDTQGFFVQAYDDNHHRLSFK------------------- 
ZmARF11  ---------------------------------------------------------------------------------------------------- 
ZmARF12  ---------------------------------------------------------------------------------------------------- 
ZmARF13  ---------------------------------------YSEMLSGYQPPNEGARNSQGFCSLPDQIAAGRPNFWHTVNAHYQ----------------- 
ZmARF14  ---------------------------------------------------------------------------------------------------- 
ZmARF15  ---------------------------------------------------------------------------------------------------- 
ZmARF16  ---------------------------------QPQNIADRSAL-LSSQILQQVQPRFQQ-IYPQNLNENKIQGHTQPEYLQVQ-QQLQRCQSFNEQKPP 
ZmARF17  ---------------------------------------------------------------------------------------------------- 
ZmARF18  ---------------------------------QPQNIAGRASPLLSSQILQQAHHQFQQQPYLQNISESTIQAQGQSEFLK---QQIQRSQSFNEQKPQ 
ZmARF19  ---------------------------------------------------------------------------------------------------- 
ZmARF20  NQLDVIINQQELKQEVGNQQRQQQPVNQAIPLSQAQTNLVQAQVIIQNQMQQQQQQQQQSPTRCQKGTSVQQLLLSQQQQDQNF-QLQQQQQLLLQQLQQ 
ZmARF21  ---------------------------------------------------------------------------------------------------- 
ZmARF22  ---------------------------------QPQNIAGRAAP-LSSQILQQAHPQFQQQPYIQNISESTIQAQGQSEFLK---QQIQRSQSFNEQKPQ 
ZmARF23  ---------------------------------------------------------------------------------------------------- 
ZmARF24  ---------------------------------------------------------------------------------------------------- 
ZmARF25  ----------------------------------------TDFKDTRSAFKDARTASQSFGDTQGFFMQAYDDSRHRLSFNNQF---------------- 
ZmARF26  ---------------------------------------------------------------------------------------------------- 
ZmARF27  -----LAQHQQQPHHVLQQQQVQPLQQSSAILQQQQAQLLQQNAIHLQQQQEQLQRQQSQPQ--QQFKATACLQSSDQHKLKEQ-QPSGGQAASQVHLLN 
ZmARF28  ---------------------------------------YNQLFSAFQPLKD-AHNP--LRPFPNKISGTRSSTWVTADARYP----------------- 
ZmARF29  -------------------------------STEAQPQLNALGIQRSDRVSCEVQTASDSNPAQEMSVKPRAPRSTDSHSSQSISDQNSKGEPRTKTRRS 
ZmARF30  ---------------------------------SPQEQCN-LNPLLQQQILQQGSQQQMVSPDAQNIQSVLNPN-------------------------- 
ZmARF31  ---------------------------------------------------------------------------------------------------- 


                   710         720        730         740        750        760         770        780         790        800         
          ....|....|....|....|....|....|....|....|....|....|....|....|....|....|....|....|....|....|....|....| 
ZmARF1   Q--QVQQSVQEQQQIKIQPFQVPNDANMVTQLSDQ-MKIQLLKALQPQ----QP--------LVMEQQKMIFDLQQQAVNSQSTAQQCSQVATQVVG-LH 
ZmARF2   -----------------------------------------------------------GGGFKRRLDAIT----PPCPISRGFVIGSAPVD-------- 
ZmARF3   -AIQQQLHQLQQMQHTHNDQK-------------------------------------------QKIQPD-------------QQYQVASSAVLPSPTSL 
ZmARF4   KKSSSHKTISDKSEISSVPSQICDKQRHGSEPT------------------------------SADFEAEQATCGNNEDSSGALTRGDFAGELQVQQ-VE 
ZmARF5   --------------------------------------------------------------------AVLTNRVLFPLLNPDLQMPPFTSPS------- 
ZmARF6   -------------PTSTLSSS-------------------------------------------SNVSFNSKNE-PSMLTSQFYWSAR------------ 
ZmARF7   -----QKLFQVTSNDARVPPW-------------------------------------------PGLSAYHADEPSSKLSCNTALCSY------------ 
ZmARF8   -------------------------------------------------------------PGLRRPDHVGPTIPIPSGISTDLTIGGAPAR-------- 
ZmARF9   LQKQQEPLPQQQQQALQQQSH-------------------------------------------QEMQHH-LSASCHDIANVASGVSESGSASQTES--- 
ZmARF10  ---NQGSTHRFADPYFYMPQQ-------------------------------------------SSVTVESSTRTQTANNDLCFWGDQNAMYGNPS-DQQ 
ZmARF11  ---LAVHNSDFTYQSVGFNES-------------------------------------------IGFSEVLQGQEISRAVPMFQGMMSEACSL------- 
ZmARF12  ---SPTGIPGFPYHCSGFGES-------------------------------------------QRFQKVLQGQEVFH--PFRGGCLADGHIR------- 
ZmARF13  --DQQGNHNLFPGSWSMMPSS-------------------------------------------TGFGMNRQSYPMIQEVGGMSQSCTNTKFGNGVYAAL 
ZmARF14  -------------PSSIFSSS-------------------------------------------LNVGFSSKNDRSTPINSHLYWTMR------------ 
ZmARF15  -------------------------------------------------------------PRLRRPDHVGPTTPIPARVSTDLTIGGAAAAARDDD--- 
ZmARF16  MHPQQQQQEPQQQQCVQTPQD-------------------------------------------QQMQEQKHLHNFHSLPDALSAFSQLSPATHSPPSAL 
ZmARF17  -----------------------------------------------------------------RLGAMT----PTPRISKGFVISSAPAS-------- 
ZmARF18  MQHQQESQQQQQPQCLPVPQH-------------------------------------------QQMQQQ-NMTNYQSISNALSPFSQLSPVSQSSPMAL 
ZmARF19  ------------------------------------------------------------HGLHRQLDHGVQVQVQQPRIAAGLIVGGHPAAR------- 
ZmARF20  QNQQQQQQQQNQQQLNKLPAQLVNLAGQQAQLSDQELQLQLLQKLQQQSLVSQPTVTLSPLQVIQEQQKLLLDMQQLSSSHSLAQQRILPQQDSDVS-LQ 
ZmARF21  -----------------------------------------------------------AHGLRHQLDHG-----ARPRIAGGLIVG-HPAAR------- 
ZmARF22  LQPQQESQQQQQSQCLQAPQH-------------------------------------------QQIQQ--NIANYQSVSNALSAFSQLSSASQSTPMAL 
ZmARF23  ---RHLNATGFAYQPLGFSES-------------------------------------------VKFSEVLQGQEMSQAVPSFMRSAFNSGTQ------- 
ZmARF24  ---TPLGSPRFSYRCSGFGES-------------------------------------------PRFQKVLQGQEVFH--PYRG-TLVDASLS------- 
ZmARF25  --QDQGSAHRFADPYFYMPQQ-------------------------------------------PSLTVESGTRTQTANNDLRFWSERNSMYGNPS-DQQ 
ZmARF26  ---LAVHSSGFTYQCVGFNES-------------------------------------------IGFSEVLQGQEISRAVPKFQGMMSEACSL------- 
ZmARF27  QILQPSSSQLQQLGLPKSPTQRPGLPSLTTMGSLQQSQLPQTPQPQQTAEYHQA--------LLQSQQPQLQQLSQSELQLQLLQKIQQQNLLSQLNPQH 
ZmARF28  --AQQANHNMLHGTLSFMPHS-------------------------------------------SGFRMIQQNNLVTPEAANFT--------GKSAFTSL 
ZmARF29  KKSSYQKTISDKSELCSVPSQICDDKKHGSEPR------------------------------LADCEAEQATCGNNEESSGALTHGDFAGELQVQQQVE 
ZmARF30  -AIQQQLQQFQQMQHAHNDQK-------------------------------------------QKIQPD-------------QPYQVPSSAVLSSPTSL 
ZmARF31  --------------------------------------------------------------------------VMAMILE---QMPPRTS--------- 


    
 
                   810         820        830         840        850        860         870        880        890         900         
          ....|....|....|....|....|....|....|....|....|....|....|....|....|....|....|....|....|....|....|....| 
ZmARF1   SSSTNQYPTQQKTQPLKT---FQDFP-GNAVSIVKPEIVTSVGARSLHVPGGVQSMKMDEVPSSSTSPSTNNNPVILRSTPS-SSKNQCLPTAAKAPQSS 
ZmARF2   ------------------------------------------ESVSCVLTIGTPRAAERSDDRKK------PHLMLFGKPILTEQQMSSR---------- 
ZmARF3   PSHLR--EKFGFSD----------------------------PNVNSSSFISSSSNENMLESNFLQVSSKSVDLSRFNQPVV-SEQQQPQ---------- 
ZmARF4   QDGLLPPPKLESSKSPDG------------------GKSVSSFPNQGCFSQFFEGLDWMIPPSCYQDSNGIHSVTTSDSIFNPSEGIPPSTMNADGMDAF 
ZmARF5   ------------------------------------------DSSEILDPESASPPNKSVRLPPAELPVQVKSIQLFGATIM------------------ 
ZmARF6   ------------------------------------------HTRADSCAASTNTVVIEKKQEPSSGGCRLFGINICSAEEEVLPEVTAPGVGYEQT--- 
ZmARF7   --------------------------------------------QTEEVAPRFSNAVEEEKKEP--GMFRLFGVNLINHARS-SATADKTSVGAGETS-- 
ZmARF8   ------------------------------------------DGVPCA--LSVGASKQNPDAVKPAG------LVLFGRTILTEHQMSLSSSGGATSPAA 
ZmARF9   -SLLSGSSYQQIYD----------------------------GNSG-----PGLHLHNGFHNCSSQESSNLLNLSRSGQFMA-SEGWPLKRLAVESLSGH 
ZmARF10  QGFNFGQNPSSWLNQPFP-----------------------QVEQPRVVRPHATVAPFDLEKTREGSGFKIFGFQVDTTSPSPVQLSSPLPAIQEHVLQT 
ZmARF11  -------------------------------------------KGGYGLHSYMRTPVAVTGLSATTQECSLTLSTPPGA--QVPSVYPDNIFNRTVVRQL 
ZmARF12  -------------------------------------------TAGMYQPDGRHVSGAAYKWSAP-QGYDFPQPAKPVFLLQESSPSSVMMFPQTRSKIT 
ZmARF13  PGRGIDRYPSGWFGHTTPG-------------------GRVDDAQPRVIKPQPLVLAHGEALKMKGNSCKLFGIHLDSPAKS-EPLKSPPSVATPAAEKW 
ZmARF14  ------------------------------------------QTRTESYSASINKAPTEKKQESATSGCRLFGIEIGS---AVSPVATVASVGQDQPP-- 
ZmARF15  -----------------------------------------DDDFPCAP-PSTGANRQKPD-AKPAG------LVLFGRTILTEQQMSRS---GATSPAA 
ZmARF16  QTVPAFSHQQNFPD----------------------------TNISSLSPSTGPSMHGMLGRLPSEAASSLPCVAMNAPVSV-SDPWSSKRVAVESVNPC 
ZmARF17  ------------------------------------------ESVSCLLTIGTPQATEKSDDIKR------PHIMLFGKPILTEQQMDSG---------- 
ZmARF18  QTILPFSQAQSFTD----------------------------TNVGSLSPSNGNTMQNTLRPFSSEAVSHLSMPRP-TAIPV-ADPWSSKRVAVESLLPS 
ZmARF19  ------------------------------------------DDVSCLLTIGTPKSKKPPSDVKKASTAAAPQLMLFGKAILTEQQISLGG-GNVVPALA 
ZmARF20  ASQAPPPMKQEQQKPSQKQFVLADVS-DVVYPQISSTNVLSKAGTQLMTPGATQSVLTEEIPSCSTSPSTATGNHLAYPIIG-RNE-HCKVTIEKVPQSS 
ZmARF21  ------------------------------------------DDISCLLTIGTAPHKKP-SDVKSAAAAPAPQLMLFGKPILTEQQISLG----FRPLPA 
ZmARF22  QTILPFSQAQSFTD----------------------------TSASSLSPSNTNTMQNTLRPFSSEAVSHLSMPRP-TAIPV-PDAWSSKRAAVESLLPS 
ZmARF23  -------------------------------------------NGRVRPFDYVQR-------SDATQGYALQQFNLPAT--EVHSPSSVLMFNQTMVPHA 
ZmARF24  -------------------------------------------NSGFHQQDGSHVPTQASKWHAQLHGCAFRGQQAPAVPSQSSSPPSVLMFQRGDPKMS 
ZmARF25  QGFSFGQNTSSWLNQPLP-----------------------QVEQSRVVRPHATVAPFDLEKTREGSGFKIFGFQVDTTNPSPVQLSSPLSAIREHVVQT 
ZmARF26  -------------------------------------------KDRYGLHSYMRAPVAVNGLSATTQECSLALSTPPGA--QVPSLYPGNVVNRTVVPQL 
ZmARF27  QSQLIQQLSQKSQEILQQQVLQHQFGGADAMGQLKHLQQTPLNHNTGSLP-PQQLVRSHSALTESEEPSSSTVPSGSRISPI-NSFSRANQGTRNLPEMP 
ZmARF28  QGHVTDQCSTGWFGSIESN-------------------SHTDHASSSLIRSQPLVIGN-DVQKTKGTSFKLFGIPLGSPEKS-EPLVSPPSVAYDG---- 
ZmARF29  QHELLAPPKIESSKSPDG------------------GKSVSSFPNQGCIPQFFEGLDWMIPPSYYQDSNGIQSVTASDNIFNPSEGIP-STMNADALDAF 
ZmARF30  PSHLR--EKFGFSD----------------------------PNVNSSSFISSSSNENMLESNFLQGSSKSVDLSRFNQPVV-SEQQ------------- 
ZmARF31  ----------------------------------------------------ASPPNNPPRPGPGQK---VNTIMLFGAT-------------------- 


                   910         920        930         940        950        960         970        980        990        1000        
          ....|....|....|....|....|....|....|....|....|....|....|....|....|....|....|....|....|....|....|....| 
ZmARF1   --VVLGSTLEQGMKPCDSTQHPMVIP-----KLAE-----ERPATGQDYMNST----QLDYLDTSSSATSVCLSQADGSLQQSFPPSSFNQHQPLREAVP 
ZmARF2   ---------GSRETLSPEATGNSSDGSVQKTGNVS----------------------------------------------------------------- 
ZmARF3   --QQQAWKQKFMGSQSMSFGGSVSLN-----SPTS-----------------------KDGSVDNKVGR------------------------------- 
ZmARF4   QTSCLSECFPNSIQEFISSPDINTLT-----FMSP----------------------EMQHLDAQHDGSNLQS--------------------------- 
ZmARF5   ---------------------------------------------------------------------------------------------------- 
ZmARF6   ---AASVELNSDKLSQPSDVNNSD---------------------------------------------------------------------------- 
ZmARF7   ----ARAAGSFEDSAQLSRVTKDHT--------------------------------------------------------------------------- 
ZmARF8   TGNSSLCWTAEKGPNVSEGSGSGSGSGVIQNSPTG----------------------------------------------------------------- 
ZmARF9   ELQPVQHKFEKVNHQSNVSHISSTLP-----PLSA-----------------------RDSYSAQASGT------------------------------- 
ZmARF10  RPSAPVNELQPVQIECLPEGSVSTAGTATEN--------------------------------------------------------------------- 
ZmARF11  GLASKFDGGA---TNAQ---QSVPFD-------------------------------------------------------------------------- 
ZmARF12  HLEYEYSRHE--DGRLDRTVPTQDMG-------------------------------------------------------------------------- 
ZmARF13  MADGIDADKSPEPHKTPKQLGATQVDPVPE---------------------------------------------------------------------- 
ZmARF14  ---ALSVDVESDQLSQPSNANKTD---------------------------------------------------------------------------- 
ZmARF15  TGNSSTCWNAEKGPNASEGSG---GSGVIQTSP-A----------------------------------------------------------------- 
ZmARF16  RPH-VSPHIEHLDMATCNMPQSSALA-----PLPG-----------------------RECLVDEDGCS------------------------------- 
ZmARF17  ---------GSREGLS------------------------------------------------------------------------------------ 
ZmARF18  RPQ-VSSQMEQLDPAPASIPHSSALA-----PLPG-----------------------RGCLVDQDVNS------------------------------- 
ZmARF19  KKSPSDDDDDVAERTVSN-SDVSSPGRSNQDGTSS----------------------------------------------------------------- 
ZmARF20  SLMSIPTAGEAVTTPIMAKELSKLNH-----NLKENVTTSKSPIVGTGHENLLNIVPSTDNLETASSATSLWPTQTDGLLHQGFP-TNFNQQQMFKDALP 
ZmARF21  PKKSPSDDAAETERTVSNNSDASSPAGTASGSTPS----------------------------------------------------------------- 
ZmARF22  RPQ-DSSQMQQLDSTPASIPHSSALA-----PLPG-----------------------RGCLVDQDANP------------------------------- 
ZmARF23  ELDGATNREE---VHGSRYLSSNAIG-------------------------------------------------------------------------- 
ZmARF24  PFEFGHFHVNKKEDRRAMFVHAGGIG-------------------------------------------------------------------------- 
ZmARF25  RPSAPVNELQPVQIECLPEVSVSTAGTAAEN--------------------------------------------------------------------- 
ZmARF26  GLARKFGGGG---TNGQ---QSGPFD-------------------------------------------------------------------------- 
ZmARF27  ----ATPHIEHLLQEIQSKTDNRIKN-----DIQG-----SKETVHAPNRHLA-----SDQLD-ASSATSFCLDESP-REGFSFPPVCLDNN-------- 
ZmARF28  -----KLQTSPTDNNEP----------------------------------------------------------------------------------- 
ZmARF29  QTSCLSECFPSSIQEFIGSPDINTLA-----FMSP----------------------EMQQLDA---GSNLPS--------------------------- 
ZmARF30  --QQQAWKQKFICSQSMSFGGSVSLN-----SPTT-----------------------KDGPVDNKIGR------------------------------- 
ZmARF31  ---------------------------------------------------------------------------------------------------- 

  


                   1010       1020       1030       1040       1050        1060       1070       1080       1090       1100        
          ....|....|....|....|....|....|....|....|....|....|....|....|....|....|....|....|....|....|....|....| 
ZmARF1   DSEFEVTDAGNNFLFGANIDGHM-EPLNEDDLLGTAFEADKYMEQMPGNGISNYISSKDSQQELSSSMIS-HPFGVADIAFN---SIDSSINDIQFLNRN 
ZmARF2   ---------------------------------------------------------------------------------DGSGSSICIGSSSRGREAS 
ZmARF3   -------DVQNQSLFSPQVDSSS-------LLYN-MVPNLT---------------SNVADNNISAFPSGSTYLQS---------PMYGCLDDS---SGL 
ZmARF4   -------TSNSYVQMSFSEESQS------ASLSGLHMEAVHIN-SSCLQPLATGSFDAGTFSKLSN-IKECQALPLQEIHNSSMGTPSCSMDAAAVEYCM 
ZmARF5   ---------------------------------------------------------------------------------------------------- 
ZmARF6   ---------------------------------------------------------------------------------------------ALAASSE 
ZmARF7   ---------------------------------------------------------------------------------------------HMVNGSP 
ZmARF8   ---------------------------------------------------------------------------------KNTSSERLPWFG-DGSSQQ 
ZmARF9   -------NDQSHLLS------SS-------FAIHDGLTAVR---------------SGGVGSGTDAITIASLRYNDMNLLPENPIATSSCLGESGTFNSL 
ZmARF10  -------------------------------------------------------------------------------------------IQQAPQSSK 
ZmARF11  ---------------------------------------------------------------------------------------------RPREIWS 
ZmARF12  ---------------------------------------------------------------------------------------------RSNQTLS 
ZmARF13  ---------------------------------------------------------------------------------------------RCPQASR 
ZmARF14  ---------------------------------------------------------------------------------------------APVASSE 
ZmARF15  ---------------------------------------------------------------------------------KAASSERPPWLGGDGSQQQ 
ZmARF16  -------DPQNHLLFGVHIDSHS-------LLMQGGIPALQ---------------NDNSSGTIPYSTSNFLSPSENDFPLNQPLRSAGCLDESDYLP-C 
ZmARF17  ----------------------------------------------------------------------------------------------QDRKAS 
ZmARF18  -------DPQNHLLFGVSIDSQS-------LLMQGGIPGLQ---------------NGNDSAAIPYSTSNFLSPSQNDFPLDHTLNSSGCLDDSGYVPPC 
ZmARF19  ---------------------------------------------------------------------------------GGGPAARACWQEEECNNRA 
ZmARF20  DVEIQEVDPTNNAFFGINSDGPLGFPMETEGLLVSAINPVKCQPNLSTDVEINYRIQKDAQQEISTSMVS-QSFGQSDIAFN---SIDSAINDGVMLNRN 
ZmARF21  ---------------------------------------------------------------------------------ISGGAPSSCQDNKAAATAT 
ZmARF22  -------DPQNHLLFGVSIDSQS-------LLMEGGIHGLQ---------------NGNDSTAIPYSTSNFLSPSQNDFPLDHTLNSSGCLDDSGYVPPC 
ZmARF23  ---------------------------------------------------------------------------------------------REAEPWP 
ZmARF24  ---------------------------------------------------------------------------------------------GTEQTTM 
ZmARF25  -------------------------------------------------------------------------------------------IQQVQQSSK 
ZmARF26  ---------------------------------------------------------------------------------------------RPRELWT 
ZmARF27  ----VQVDPRENFLIAENVDTLM-----PDALLSRGMSSGKGICNLP----SGQRDHRDVENELSSAAFSSQSFGVPDMSFKPGCSSDVAVADGGMASQG 
ZmARF28  ----------------------------------------------------------------------------------------------CSEATQ 
ZmARF29  -------TSNSYVQMSFSEESHS------ASLSGLHMEAIHINSSSCSQPLATGSFDAGAFPKLSN-IRECQPLPLQEIHTSSMGTPSCSMDAAA-EYGT 
ZmARF30  -------DVQNQTLFSPQVDSSS-------LLYN-MVPNLT---------------SNVADNNISTIPSGSTYLQS---------PMYGCLDDS---SGL 
ZmARF31  ---------------------------------------------------------------------------------------------------- 

 

                                               Ⅲ                                 Ⅳô


                   1110       1120       1130       1140       1150       1160        1170       1180       1190       1200        
          ....|....|....|....|....|....|....|....|....|....|....|....|....|....|....|....|....|....|....|....| 
ZmARF1   SRAPGPVQQRMRTYTKVHKRG-AVGRSIDINRYSGYDELKHDVARMFGIEGQLSDQNRVGWKLVYEDHEKDVLLVGDDPWEDFVNCVRCIRILSPQEERQ 
ZmARF2   RLGF----EFEAGHCKVFVESEDVGRTIDLSVFGSYEELYGQLADMFGIEKAEVMSHLCYRD-----AAGAVKRTGDEPFCDFMKVARRLTIVESTEGRL 
ZmARF3   LQNTGENDPTTRTFVKVYKSG-SVGRSLDITRFSNYAELREELGQMFGIKGQLDDPDRSGWQLVFVDRENDVLLLGDDPWESFVNSVWYIKILSPEDVHK 
ZmARF4   DRSVKPLKPPVRTYTKVQKLG-SVGRSIDVTRFRDYHELRSAIACMFGLQGKLEHPGSSDWKLVYVDYENDVLLVGDDPWEEFINCVRCIRILAPSEVQQ 
ZmARF5   ---------------VHVVRSVACG--------GCCEELNGAPSGDATADG-SVGKDV------------------------------------------ 
ZmARF6   RSPLESQSRQVRSCTKVIMQGMAVGRAVDLTKLSGYSDLCQKLEEMFDIQGELGSTLK-KWRVIFTDDEDDMMLVGDDPWDEFCRMVKRIYIYTYEEAKK 
ZmARF7   REIQSHQSCSGRSRIKVQMHGNDVCRAVDLGNLDGYEQLMGEVGEMFEIKDLGSKEKE-EWKVTFINDENETMEVGAVPWQEFCQMVRKIVIHSIGDRGH 
ZmARF8   ASE----PGLEPGQCKVFVESDTVGRNLDLSALGSFDELYGRLSEMFGVEGAEMRSRVLYRG-----ATGEVRHAGDEPFSDFVKSARRITILTDAGSDN 
ZmARF9   DDVCGVNPSQGGTFVKVYKSG-SPGRSLDITRFSSYYELRSELERLFGLEGQLEDPVRSGWQLVFVDRENDILLVGDDPWQEFVNSVWCIKILSPQDVQQ 
ZmARF10  DIQSKSQGASTRSCTKVHKQGVALGRSVDLSKFTDYGELQAELDKMFDFEGELVSGSQ-NWQIVYTDDEGDMMLVGDDPWEEFCSIVRKIYIYTKEEVQK 
ZmARF11  KPQHEISDQTKMDHFETRRASAPGDDAAKHGSGGEVVRKTSCRLFGFSLTEKILPADDDGIKEVTYE-------PECQNPRMLDLFGYNCSAPSAALPAL 
ZmARF12  LWPHLVSGEAIEECTGTVNMHSP-VSGAEHESNNESTVENGCKIFGISLAEKIRSCDEADSCSAKCN--SRLQPLKSQMPKSLGSCWATVHEQRPVVGRV 
ZmARF13  GTQCKSQGGSTRSCKKVHKQGMALGRSVDLTKFNGYTELVAELDEMFDFNGELKGCSK-EWMVVYTDYEGDMMLVGDDPWNEFCSMVHKIFVYTREEVQR 
ZmARF14  RSLNESESRQVRSCTKVIMQGMAVGRAVDLTRLDGYADLHRKLEEMFDIQGELSANLK-KWKVIYTDDEDDTMLVGDDPWNEFLRMVKRIYIYSYEEAKS 
ZmARF15  QASGELGLGLEPGQCKVFVESDTVGRNLDLAALRSFDELYGRLSGMFGVAGAELRSRVLYRG-----A------AGDEPFSDFVRSARRLTVLTDAGSDN 
ZmARF16  AENAEQANQQFATFVKVYKSG-TVGRLLDITRFSSYDELRSEVGRLFGLEGQLEDPLRSGWQLVFVDREDDVLLVGDDPWQEFVNSVSCIKILSPEEVQR 
ZmARF17  ELG------LEDGHCKVFMESEDVGRTIDLSVFGSYEELYGQLADMFGIEKAEIMRHLCYRD-----AAGAVRHTGEEPFNDFMKVARRLTIIEGTEGRP 
ZmARF18  SDNSDKVNRPPATFVKVYKSG-TYGRSLDITRFSSYHELRRELGRLFGLEGQLEDPLRSGWQLVFVNREEDVLLVGDDPWQEFVSTVSCIKILSPQEVQQ 
ZmARF19  AGSE----DDLLGHCKVFMQSEDVGRTLDLSAVASYEELYQRLADMFGVDKAELTSHVFYRDD----ASGALKHPGDEPFSEFTKTARRLTILTDESSDS 
ZmARF20  SCPPAPPQ-RMRTFTKVYKRG-AVGRSIDIGRFSGYEELKHAVARMFGIEGQLEDRQRIGWKLVYTDHEDDVLLLGDDPWEEFVNCVKCIRILSPQEVQQ 
ZmARF21  ATDD----DDLLGHCKVFMQSEDVGRTLDLSAVASYEELYQRLADMFGVDRAELTSHVFYRDG----ASGALKHAGDEPFSEFTKTARRLTIQTDAR--- 
ZmARF22  SDNSDQVNRPPATFVKVYKSG-TYGRSLDITRFSSYHELRRELGRLFGLEGQLEDPLRSGWQLVFVDREEDVLLVGDDPWQEFASTVSCIKILSPQEVQQ 
ZmARF23  SMQQQRASVNGSEPLDTTEASAP-ARNAESGSVGRGAGRSNCKLFGFSLTEKILGTDGGGVKEGNYE-------VDRQTPRVLDLFGHGSTP--GALHAL 
ZmARF24  LQAHHVSGGTGNRDVTVEKSHPA-VAAASD---NREVSKNSCKIFGISLTDKVPAMKEKGCGDINTNYPSPFLFLKQQVPKSLGNSCATVHEQRPVVARV 
ZmARF25  DIQSKSQGASTRSCTKVHKQGVALGRSVDLSKFTDYGELKAELDKMFEFEGELVSANR-NWQIVYTDNEGDMMLVGDDPWEEFCNIVRKIYIYTKEEVQK 
ZmARF26  RPQHETPDQMNLDQFETRRPSVP-VDAAKLGSGGGEVRKTSCRLFGFSLTEKILPADDDDVKEVSYE-------TECQNPRMLDLFGYNCSTPSAALPAL 
ZmARF27  LWN--SQTQRMRTFTKVQKRG-SVGRSIDITRYRGYEDLRHDLACMFGIQGQLEDPYRTDWKLVYVDHENDILLVGDDPWEEFVSCVKSIKILSSVEVQQ 
ZmARF28  NIQNKVQSSSTRSCKKVHKQGSALGRSIDLTKFACYDELIAELDQMFDFDGELKSPCR-NWLVVYTDNEGDMMLVGDDPWNEFCDMVHKIFIYTREEVER 
ZmARF29  DRSAKQMKPPVRTYTKVQKLG-SVGRCIDVTRFRDYHELRSAIACMFGLQGKLEHPGSSDWKLVYVDYENDVLLVGDDPWEEFINCVRCIRILSPSEVQQ 
ZmARF30  LQNTGENDPTTRTFVKVYKSG-SVGRSLDITRFSNYAELREELGQMFGIKGQLDDPDRSGWQLVFVDRENDVLLLGDDPWESFVNSVWYIKILSPEDVHK 
ZmARF31  ---------------IHVVQSDTDD--------DGYEQLNRDPPPNAAGGGDDMDHDSAENI-------------------------------------- 


  
           Ⅳô
       

                   1210       1220       1230       1240       1250       1260    
          ....|....|....|....|....|....|....|....|....|....|....|....| . 
ZmARF1   MRLAS--------------DYGDSFLGNQACSSSDGGRPWRVTGD--------------- 
ZmARF2   QKPLVEYMA--------------------------------------------------- 
ZmARF3   MGKPG-----------------------NDPRYLS------------------------- 
ZmARF4   MSENG-------------------VHVLNDCMQMA------------------------- 
ZmARF5   ------------------------------------------------------------ 
ZmARF6   LTSKSKLPVS-----SDSSKLSAANSLSE------------------------------- 
ZmARF7   MEACP--------------CLGQDGKRDY------------------------------- 
ZmARF8   LGS--------------------------------------------------------- 
ZmARF9   MVRGGGDLLSTTGVRTLQGSVCDDYSAGHDMQNLTGSIAPVVPLDY-------------- 
ZmARF10  MNSKSAAPRKEESPAAGGGGGCAAATNE-------------------------------- 
ZmARF11  CAAP-FGM---------------------------------------------------- 
ZmARF12  VDVSATDM---------------------------------------------------- 
ZmARF13  MNPGALNSRPEDSGLANSTERGSASTAAAREAPGYQSASSLNSDNC-------------- 
ZmARF14  LTRKAKPPVVGDTIKPDPNKLPPESDVPHDSNNNAPVAADYQD----------------- 
ZmARF15  LGS--------------------------------------------------------- 
ZmARF16  MGKPGIQLLSSAP-SRRLGNGCDSYASMQEPRGLDAGMAPVGSVEF-------------- 
ZmARF17  QKPLVEYMVERA------------------------------------------------ 
ZmARF18  MGKQGLELLSSAP-ARRLGSSCDDYVSRQESRSLSTGIASVGSVELG------------- 
ZmARF19  LAR--------------------------------------------------------- 
ZmARF20  MSLDG--------------DLGNNVLSNQACSSSDGGNAWKPRRDQNPGNPSIGFYDQFE 
ZmARF21  ------------------------------------------------------------ 
ZmARF22  MGKQGLELLSSAPAARRLGSSCDGYVSRQESRSLSTGIASVGSVEF-------------- 
ZmARF23  CAAP-LGI---------------------------------------------------- 
ZmARF24  IDVSTVDMMI-------------------------------------------------- 
ZmARF25  MNSKSSVPRKEEPPAAG-EGCAAAAANE-------------------------------- 
ZmARF26  CAAAPFGM---------------------------------------------------- 
ZmARF27  MSLDG--------------DLG-CIPQTQACSASDDANAWRA------------------ 
ZmARF28  MNPGALNARLEDC-LSDSLGRGLAS-KEPRSGPSTSAVDSENRANLSSQ----------- 
ZmARF29  MSENG-------------------VHVLNDCIQIA------------------------- 
ZmARF30  MGKPG-----------------------NDPRYLS------------------------- 
ZmARF31  ------------------------------------------------------------ 
