# Supplementary material for: Genome-wide identification and expression profiling of auxin response factor (ARF) gene family in maize
Source: BMC Genomics. 2011 Apr 7;12:178. doi: 10.1186/1471-2164-12-178 (PMC3082248; doi:10.1186/1471-2164-12-178)
Supplement: Additional file 4 — miR160, 167 and TAS3 target site prediction for ZmARF genes. [file 1471-2164-12-178-S4.DOC]

**Additional file 4. *miR160*, *167* and *TAS3* target site prediction for *ZmARF* genes**

(A)

*ZmARF2* 1309bp GCAGGCAUACAGGGAGCCAGGCAU 1332bp

*ZmARF5* 1186bp GCUAGCAUGCAGGGAGCCAGGCAC 1209bp

*ZmARF8* 1352bp –CAGCCAUACAGGGAGCCAGGCAU 1374bp

*ZmARF15* 1370bp -CAGCCAUACAGGGAGCCAGGCAU 1392bp

*ZmARF17* 1324bp GCAGGCAUACAGGGAGCCAGGCAU 1347bp

*ZmARF19* 1348bp GCAGGCAUACAGGGAGCCAGGCAU 1371bp

*ZmARF21* 1330bp GCAGGCAUACAGGGAGCCAGGCAU 1353bp

:|:||||||||||||||||||

*zma-miR160a* 3’---ACCGUAUGUCCCUCGGUCCGU---5’

(B)

*ZmARF3* 2281bp GAUAGAUCAGGCUGGCAGCUUGUAU 2305bp

*ZmARF9* 2362bp GUAAGAUCAGGCUGGCAGCUUGUAU 2386bp

*ZmARF16* 2449bp CUGAGAUCAGGCUGGCAGCUUGUAU 2473bp

*ZmARF18* 2470bp UUGAGAUCAGGCUGGCAGCUUGUAU 2494bp

*ZmARF22* 2506bp CUGAGAUCAGGCUGGCAGCUUGUAU 2530bp

*ZmARF30* 2260bp GAUAGAUCAGGCUGGCAGCUUGUAU 2284bp

:||||||:|||||||||||

*zma-miR167a* 3’---AUCUAGUACGACCGUCGAAGU---5’

(C)

*ZmARF11* 1200bp -----------UGGAAAGUUUCCCAGGGUCUUGCAAGGUCAAGAUUUGAU..170bp..UCUCAGAGGUCUUGCAAGGUCAAGAAAUUUCUCGGGCAGUUCCUAUGUUCCAAGGAAUGAUGUCUGAGGCUUGU----------- 1500bp

*ZmARF12* 1250bp -------GAUCUGCCAGAUUCCACAAGGUCUUGCAAGGUCAAGAAUUGUU..170bp..UCCAAAAGGUCUUGCAAGGUCAAGAAGUGUUUCA---------UCCUUUCCGAGGAGGAUGUUUGGCUGAUGGCCAUAUAAGAAC 1550bp

*ZmARF23* 1200bp --AGUGGAAACUGAACGUUUCCACAGGGUCUUGCAAGGUCAAGAAUUGGU..170bp..UCUCAGAGGUCUUGCAAGGUCAAGAAAUGUCUCAGGCGGUUCCUUCCUUCAUGAGAUCUGCUUUC-------------------- 1500bp

*ZmARF24* 1300bp UUUGCGGAAUCUGCCCAAUUCCACAAGGUCUUGCAAGGUCAAGAAUUACU..170bp..UCCAAAAGGUCUUGCAAGGUCAAGAAGUAUUUCA---------UCCCUACAGAGGAACUC------------------------- 1600bp

*ZmARF26* 1200bp -----GGAAACUGGAAAGUUACCCAGGGUCUUGCAAGGUCAAGAAUUGAU..170bp..UCUCAGAGGUCUUGCAAGGUCAAGAAAUUUCUCGGGCGGUUCCUAAGUUCCAAGGAAUGAUGUCGGAA----------------- 1500bp

:|||:|||:|||||||||||| ||||:|||:||||||||||||

*zma-TAS3*  3' CUCCGGAAUGUUCCAGUUCUU 5' 3' CUCCGGAAUGUUCCAGUUCUU 5'

|||||||||||||||||||||

*pre-tasiR-ARF*(RC) 603bp **-----AGGAUGCAGACACAGAGUGAGAGCCUUACAAGGUCAAGAA-------------------GAGGCCUUACAAGGUCAAGAA---CGUUAAUUCUGGUAUGAGAAACCAGAAAACAGACAUACGAGUCAACCUCUGGUGGA** 487bp

(A) Sequences of miR160 target sites on *ZmARF* mRNAs. The Watson-Crick base pairings to the *zma-miR160a* sequence are shown.

(B) Sequences of miR167 target sites on *ZmARF* mRNAs. The Watson-Crick base pairings to the *zma-miR167a* sequence are shown.

(C) Alignment of maize tasiR-ARF sequences and the potential *ZmARF* target genes. To visualize similarity, the sense orientation of each target gene sequence was aligned with the reverse complement (RC) of the maize *pre-tasiR-ARF* sequences (pc0085991). The (-) character marks the gap opened to allow the closest alignment between the multiple sequences of *ZmARF*s. Nucleotides of maize *pre-tasiR-ARF* are boldfaced. The regions of sequence similarity between the 21-nt *pre-tasiR-ARF* (shaded with gray) and the ARF target genes are boxed. The potential target gene mRNAs aligned more closely with the *zma-TAS3* (red and blue letters), with two to three mismatches. The target gene name and the location of the recognition sequence are indicated (note that all of maize genes, with two *zma-TAS3* recognition sites).
