# Supplementary material for: Genome-wide identification and expression profiling of auxin response factor (ARF) gene family in maize
Source: BMC Genomics. 2011 Apr 7;12:178. doi: 10.1186/1471-2164-12-178 (PMC3082248; doi:10.1186/1471-2164-12-178)
Supplement: Additional file 6 — The relative expression levels of 31 ZmARFs in three tissues of maize inbred line B73. Leaves (8-day-old seedling), roots (8-day-old seedling) and embryos (15d after pollination) of maize inbred line B73 were used for real-time RT-PCR analysis. [file 1471-2164-12-178-S6.DOC]

**Additional file 6. The relative expression levels of 31 *ZmARFs* in 3 tissues of maize inbred line B73**

| Gene | Leaves ^a^ | Mean Ct ^b^ | Roots ^c^ | Mean Ct ^b^ | Embryos ^d^ | Mean Ct ^b^ |
| --- | --- | --- | --- | --- | --- | --- |
| *ZmARF1* | 12.83±1.08 | 28.53 | 40.21±3.75 | 26.88 | 0.47±0.01 | 33.30 |
| *ZmARF2* | 3.13±0.26 | 30.57 | 2.09±0.11 | 31.15 | 8.28±1.01 | 29.16 |
| *ZmARF3* | 0.18±0.08 | 34.69 | 1.23±0.12 | 31.92 | 1.91±0.11 | 31.28 |
| *ZmARF4* | 0.01±0.00 | 38.86 | 8.06±0.55 | 29.20 | 47.14±7.28 | 26.66 |
| *ZmARF5* | 1.00±0.18 | 32.21 | 1.32±0.29 | 31.81 | 4.47±0.41 | 30.05 |
| *ZmARF6* | 3.37±0.67 | 30.46 | 5.60±0.40 | 29.73 | 12.75±0.36 | 28.54 |
| *ZmARF7* | 0.28±0.03 | 34.05 | 7.04±0.23 | 29.40 | 0.28±0.04 | 34.05 |
| *ZmARF8* | 1.54±0.18 | 31.59 | 7.58±0.59 | 29.29 | 61.87±5.51 | 26.26 |
| *ZmARF9* | 2.21±0.12 | 31.07 | 6.46±0.28 | 29.52 | 0.09±0.00 | 35.69 |
| *ZmARF10* | 1.96±0.11 | 31.24 | 2.17±0.31 | 31.10 | 20.63±2.55 | 27.85 |
| *ZmARF11* | 3.81±0.52 | 30.28 | 0.83±0.11 | 32.48 | 2.24±0.05 | 31.05 |
| *ZmARF12* | 2.91±0.07 | 30.67 | 3.08±0.47 | 30.59 | 6.75±0.57 | 29.46 |
| *ZmARF13* | 7.44±1.25 | 29.32 | 1.09±0.13 | 32.09 | 5.16±0.41 | 29.85 |
| *ZmARF14* | 1.95±0.09 | 31.25 | 2.25±0.15 | 31.04 | 1.89±0.07 | 31.30 |
| *ZmARF15* | 6.89±1.04 | 29.43 | 7.84±0.99 | 29.24 | 14.70±0.74 | 28.34 |
| *ZmARF16* | 1.27±0.16 | 31.87 | 40.47±2.65 | 26.88 | 1.32±0.09 | 31.81 |
| *ZmARF17* | 1.01±0.05 | 32.20 | 2.51±0.15 | 30.89 | 1.93±0.13 | 31.27 |
| *ZmARF18* | 0.03±0.01 | 37.27 | 0.02±0.00 | 37.86 | 0.55±0.00 | 33.08 |
| *ZmARF19* | 6.70±0.87 | 29.47 | 5.15±0.47 | 29.85 | 1.95±0.10 | 31.25 |
| *ZmARF20* | 0.86±0.03 | 32.43 | 6.20±0.84 | 29.58 | 0.91±0.01 | 32.35 |
| *ZmARF21* | 21.26±1.30 | 27.80 | 9.67±0.89 | 28.94 | 1.16±0.16 | 32.00 |
| *ZmARF22* | 0.86±0.04 | 32.43 | 0.16±0.02 | 34.86 | 0.34±0.00 | 33.77 |
| *ZmARF23* | 0.33±0.05 | 33.81 | 27.90±1.10 | 27.41 | 0.93±0.05 | 32.32 |
| *ZmARF24* | 13.57±1.38 | 28.45 | 6.13±0.58 | 29.60 | 4.10±0.24 | 30.18 |
| *ZmARF25* | 2.11±0.09 | 31.14 | 1.41±0.18 | 31.72 | 0.19±0.07 | 34.61 |
| *ZmARF26* | 2.55±0.45 | 30.86 | 4.45±0.17 | 30.06 | 13.90±1.06 | 28.42 |
| *ZmARF27* | 10.25±0.39 | 28.86 | 5.10±0.22 | 29.86 | 0.62±0.00 | 32.90 |
| *ZmARF28* | 1.81±0.21 | 31.36 | 0.87±0.10 | 32.41 | 16.29±0.91 | 28.19 |
| *ZmARF29* | 0.01±0.00 | 38.86 | 8.87±0.73 | 29.06 | 19.81±2.02 | 27.91 |
| *ZmARF30* | 0.12±0.03 | 35.27 | 2.65±0.18 | 30.81 | 1.27±0.23 | 31.87 |
| *ZmARF31* | —— |  | —— |  | 1.29±0.04 | 31.85 |

^a^ Leaves of 8-day-old seedlings.

^b^ Average Ct values of quantitative real-time PCR.

^c^ Roots of 8-day-old seedlings.

^d^ Embryos of 15 days after pollination.
