# Supplementary material for: Genome-wide identification and expression profiling of auxin response factor (ARF) gene family in maize
Source: BMC Genomics. 2011 Apr 7;12:178. doi: 10.1186/1471-2164-12-178 (PMC3082248; doi:10.1186/1471-2164-12-178)
Supplement: Additional file 7 — Primers used for stability evaluation of housekeeping gene expression. [file 1471-2164-12-178-S7.DOC]

**Additional file 7. Primers used for stability evaluation of housekeeping genes**

| **Symbol** | **GenBank ID** | **Name** | **Forward primer (5′-3′)** | **Reverse primer (5′-3′)** |
| --- | --- | --- | --- | --- |
| *ACTIN* | EU960271 | *β-Actin* | CAATGGCACTGGAATGGT | ATCTTCAGGCGAAACACG |
| *GAPDH* | EU963991 | Glyceraldehyde-3-phosphate dehydrogenase | CCACATGAACAGCACCAAGT | GTCACTCAATGCTCAACCAA |
| *18S* | AF168884 | 18S ribosomal RNA | TGACGGAGAATTAGGGTTCG | CCTCCAATGGATCCTCGTTA |
| *TUB* | NM_001111998 | Tubulin | ACACCACCATTGGGAGTCTA | TTGTGGGGACCACTACTTTC |
| *UBQ* | U29159 | Ubiquitin | AGACCCTGACTGGAAAAACC | CGACCCATGACTTACTGACC |
