# Supplementary figures and images for: Genome-wide identification and expression profiling of auxin response factor (ARF) gene family in maize
Source: BMC Genomics. 2011 Apr 7;12:178. doi: 10.1186/1471-2164-12-178 (PMC3082248; doi:10.1186/1471-2164-12-178)

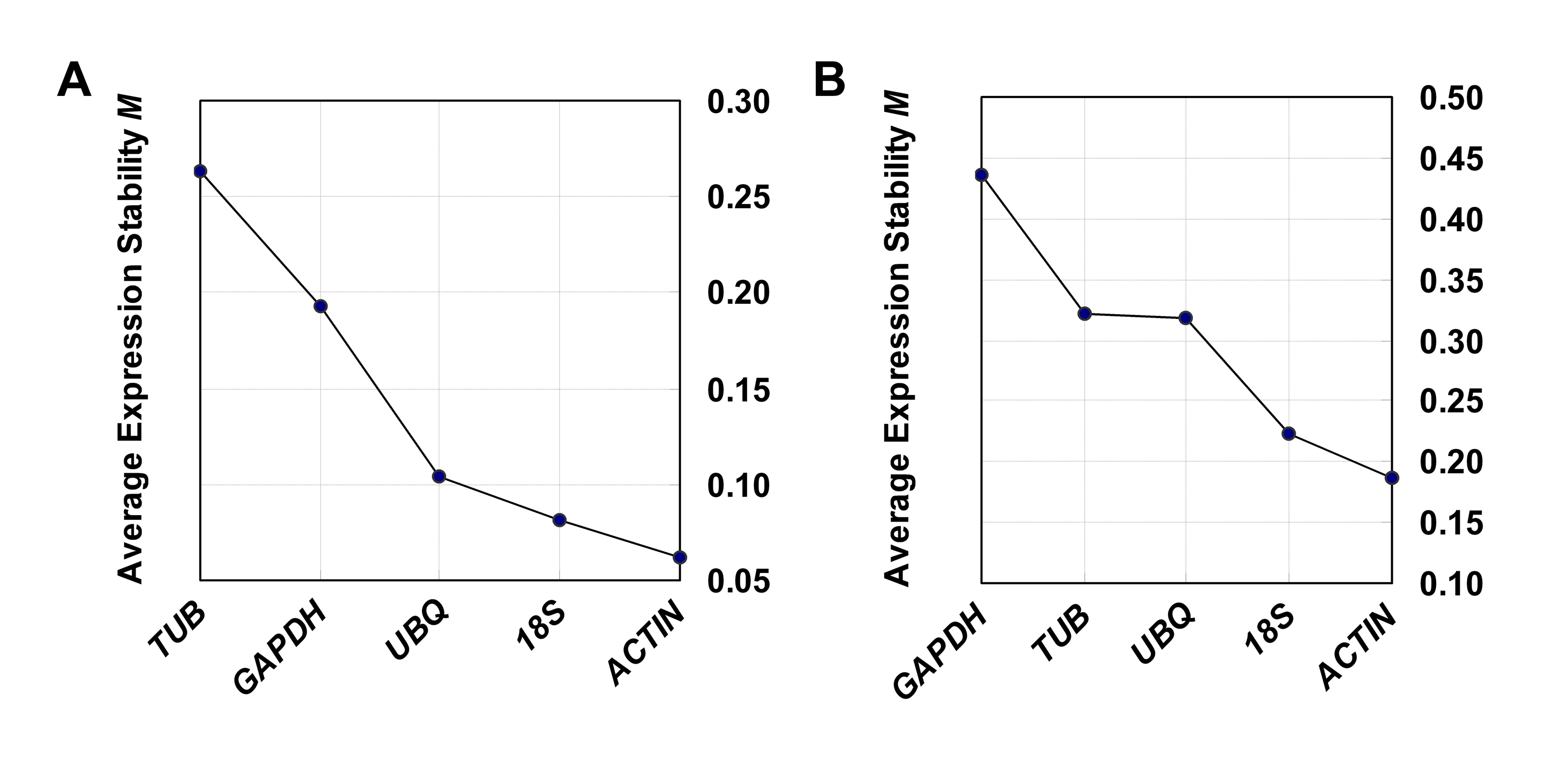

Supplement: Additional file 8 — Stability evaluation of housekeeping gene expression. Auxin treated 8-day-old primary roots (A) and embryos during seed development and germination (B) were used for evaluating the stability of five housekeeping genes. [file 1471-2164-12-178-S8.JPEG]
