# Supplementary material for: Genome-wide identification and expression profiling of auxin response factor (ARF) gene family in maize
Source: BMC Genomics. 2011 Apr 7;12:178. doi: 10.1186/1471-2164-12-178 (PMC3082248; doi:10.1186/1471-2164-12-178)
Supplement: Additional file 9 — Primers used for expression study of ZmARF genes. [file 1471-2164-12-178-S9.DOC]

**Additional file 9. Primers used for expression study of *ZmARF* genes**

| Genes | Forward primer (5′-3′) | Reverse primer (5′-3′) |
| --- | --- | --- |
| *ZmARF1* | GCCTGAGCACAAATGTTTGATA | CACATCGAAGAGGCTACATCAG |
| *ZmARF2* | TGGCTTGATTGCATGACTGT | CCAAGTCACGACAGACGAAA |
| *ZmARF3* | GGAAACGATCCACGCTACCT | TACAAGCGACGGGGAGTTC |
| *ZmARF4* | GCCTTCGGAAGTACAGCAGAT | GCGTGGCTACGAAGAACACT |
| *ZmARF5* | AGATGTTTAAACCATGCACCTG | CAACCAACCAACAATATGAAGTG |
| *ZmARF6* | TGATTATGGGGGTGAATGCT | CAAACCAGGGACCTGCTAAA |
| *ZmARF7* | GTGCTGCTGAAACATGATGG | CGCTAAGGCTTTAGGCAGAT |
| *ZmARF8* | CAAGTCGGCACGGAGGAT | CTTTTCTCCTCCCTCACTGC |
| *ZmARF9* | GACCGAGATGAAGACCAGAAAC | ACAGGGCTAGGTCACCTCTACA |
| *ZmARF10* | GCCAAGGAAGGAGGAATCTC | CACAGATATTTACACCGGAAGG |
| *ZmARF11* | TGCTACTTCAATAACGCTGCTG | CTGAAGGATCAGCCACAGAA |
| *ZmARF12* | CTGATCCACATGGCATATCCT | TTGCCATAACGCACACCTTA |
| *ZmARF13* | CGTCGCTTAACTCCGACAAC | AGGCATTAAGCTCCGCTACA |
| *ZmARF14* | CCTTTTCTTTCCCCTCTTGG | ACACACGGAACGATACCAGT |
| *ZmARF15* | GTGCAAGGTGTTCGTGGAGT | CTCCCAGCATCGGTCAATAC |
| *ZmARF16* | CTCCCCGTCTGATGATGTCT | GGTTTTACTGCATCGCAACA |
| *ZmARF17* | TCGTGGAATACATGGTCGAG | ACGAAGCGTACACCAAGGTT |
| *ZmARF18* | GTTGGTCTGCGACTTGATGA | AGAGCATAGGCCTGGCTACA |
| *ZmARF19* | GCGACAGCTTAGCGAGGTAG | GCCACTTGAACTGACAGCAG |
| *ZmARF20* | TACGACCAATTTGAGTGACCTG | TTTCGACTCAGCAACAGCTCTA |
| *ZmARF21* | GTAGAAGAAGGCGGCAGACA | GATAAGAAACGACCGACACGA |
| *ZmARF22* | TAGTTTAGGGCTGGCCTGTG | CGGCTGCTGGTAGTTTCTTC |
| *ZmARF23* | CCGGGTGCTAGACTTGTTTG | TGCATGGATCTCAAGAGAGG |
| *ZmARF24* | TGTTGCTAGGGTGATTGACG | TGTTGCTAGGGTGATTGACG |
| *ZmARF25* | GGTCCAGAAGATGAACTCGAA | CCCATTCACCACCACTGTT |
| *ZmARF26* | TGATGCTGCTTCGAATCTTT | TTCTGAAGGATCATCGACGTAA |
| *ZmARF27* | CAAGCCTGTAGTGCTTCTGATG | GAGCTTCTCATCGACTCTGTCA |
| *ZmARF28* | GAGAACCGCGCTAACTTGTC | CTTTTACCCGGAGGCATACA |
| *ZmARF29* | TGCATCCGGATCTTATCACC | GGCAGGATGTTGACATCTGTAAT |
| *ZmARF30* | GATCCCCGCTACCTATCTTAAA | CAAGTAACGGGGGAGTTCTG |
| *ZmARF31* | CCGACGATGATGGTTATGAA | GACTCGTTTTATTTTTGCATCC |
